# Pyroptosis Remodeling Tumor Microenvironment to Enhance Pancreatic Cancer Immunotherapy Driven by Membrane Anchoring Photosensitizer

**DOI:** 10.1002/advs.202202914

**Published:** 2022-08-18

**Authors:** Meng Wang, Min Wu, Xingang Liu, Shiyi Shao, Junmin Huang, Bin Liu, Tingbo Liang

**Affiliations:** ^1^ Department of Hepatobiliary and Pancreatic Surgery First Affiliated Hospital Zhejiang University School of Medicine Hangzhou 310003 P. R. China; ^2^ Department of Chemical and Biomolecular Engineering National University of Singapore 4 Engineering Drive 4 Singapore 117585 Singapore; ^3^ Joint School of National University of Singapore and Tianjin University International Campus of Tianjin University Binhai New City Fuzhou 350207 P. R. China; ^4^ Zhejiang Provincial Key Laboratory of Pancreatic Disease Hangzhou 310003 P. R. China; ^5^ Innovation Center for the Study of Pancreatic Diseases Hangzhou 310003 P. R. China; ^6^ Zhejiang Provincial Clinical Research Center for the Study of Hepatobiliary & Pancreatic Diseases Hangzhou 310003 P. R. China; ^7^ Cancer Center Zhejiang University Hangzhou 310058 P. R. China; ^8^ Research Center for Healthcare Data Science Zhejiang Lab Hangzhou 310003 P. R. China

**Keywords:** pyroptosis, photodynamic therapy, aggregation‐induced emission, immunotherapy, pancreatic cancer

## Abstract

Immunotherapy, the most promising strategy of cancer treatment, has achieved promising outcomes, but its clinical efficacy in pancreatic cancer is limited mainly due to the complicated tumor immunosuppressive microenvironment. As a highly inflammatory form of immunogenic cell death (ICD), pyroptosis provides a great opportunity to alleviate immunosuppression and promote systemic immune responses in solid tumors. Herein, membrane‐targeted photosensitizer TBD‐3C with aggregation‐induced emission (AIE) feature to trigger pyroptosis‐aroused cancer immunotherapy via photodynamic therapy (PDT) is applied. The results reveal that pyroptotic cells induced by TBD‐3C could stimulate M1‐polarization of macrophages, cause maturation of dendritic cells (DCs), and activation of CD8^+^ cytotoxic T‐lymphocytes (CTLs). Pyroptosis‐aroused immunological responses could convert immunosuppressive “cold” tumor microenvironment (TME) to immunogenic “hot” TME, which not only inhibits primary pancreatic cancer growth but also attacks the distant tumor. This work establishes a platform with high biocompatibility for light‐controlled antitumor immunity and solid tumor immunotherapy aroused by cell pyroptosis.

## Graphical abstract

A single effective light‐induced pyroptosis activates cascade amplification of anti‐tumor immunity for pancreatic cancer therapy by membrane anchoring photosensitizer with aggregation‐induced emission feature.

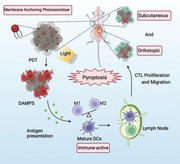

## 1 Introduction

Pancreatic cancer is one of the leading causes of cancer mortality with a five‐year survival rate of at most 10%.^[^ ^1^ ^]^ Despite advances in diagnosis, surgical, and medical therapies, pancreatic cancer has been suffering from extremely poor prognosis and high lethality.^[^ ^1^ ^]^ The particular challenge of pancreatic cancer treatment is that it is naturally resistant to conventional radiotherapy, chemotherapy, and other locoregional therapies.^[^ ^2^ ^]^ Immunotherapy, which aims to recruit immune cells for tumor cell recognition and ablation, has recently become a much‐anticipated cancer treatment strategy.^[^ ^3^ ^]^ Currently, various clinical trials endeavor to validate the efficiency of immunotherapeutic approaches for pancreatic cancer, including adoptive T‐cell therapy (ACT), immune checkpoint inhibitors (ICIs), and cancer vaccines.^[^ ^4^ ^]^ ACT is applied as a personalized therapy relying on the ex vivo modification of patients’ tumor‐specific lymphocytes with high activity, and the subsequent administration back to the autologous host. Though with great potential to induce an antitumor immune response, the widespread application of ACT would be hindered by the challenges of technology scaling‐up and standardization.^[^ ^5^ ^]^ ICIs activate antitumor immune responses by inhibition of immune checkpoint molecules such as programmed cell death protein‐1 (PD‐1), programmed cell death protein ligand‐1 (PD‐L1), and cytotoxic T‐lymphocyte‐associated antigen 4 (CTLA‐4). However, the utilization of ICIs is significantly limited since only about one‐third of cancer patients show positive responses to ICI agents.^[^ ^6^ ^]^ Meanwhile, several vaccine‐based strategies have also been applied in pancreatic cancer including deoxyribonucleic acid (DNA), peptide, and whole‐cell vaccines which activate the antitumor immune responses by presenting tumor antigens to the host immune system. However, challenges in vaccine manufacturing and limitations of immune response identification seriously hindered the development of cancer vaccine.^[^ ^7^, ^8^ ^]^ Besides, the complex TME of pancreatic cancer, which consists of an abundance of blood vessels, fibroblasts, pancreatic stellate, and immune cells, is the most vital obstacle to efficient immunotherapy.^[^ ^9^, ^10^ ^]^ Thus, an alternative strategy is highly desirable for more efficient immunotherapy of pancreatic cancer.

Different from apoptosis which is usually regarded as an immune‐tolerogenic process, pyroptosis is a highly inflammatory programmed cell death (PCD), providing a great opportunity to alleviate immunosuppression and promote systemic immune responses toward solid tumors.^[^ ^11^, ^12^ ^]^ Activation of inflammatory caspases to cleave gasdermin‐D (GSDMD) is an important event for pyroptosis, which releases gasdermin‐D *N* terminal domain (N‐GSDMD) to translocate and further drill pores on the cell membrane to induce cell swelling, membrane disruption, and immunostimulatory release of cellular contents. Pyroptosis ultimately triggers ICD, and further converts immunosuppressive “cold” TME to immunogenic “hot” TME with massive tumor‐infiltrating lymphocytes.^[^ ^13^ ^]^ Besides, pyroptotic cells can not only play the role of tumor‐associated antigens (TAAs) via releasing cell contents including cytokines and proinflammatory factors but also emit danger signals in the form of danger‐associated molecular patterns (DAMPs), acting as immune adjuvants necessary for the recruitment and maturation of antigen‐presenting cells (APCs), leading to stronger immune activation.^[^ ^14^ ^]^ In recent studies, chemotherapeutic drugs such as decitabine (DAC),^[^ ^15^ ^]^ iron oxide,^[^ ^16^ ^]^ genic hemicyanine (CyNH2),^[^ ^17^ ^]^ and glucose oxidase^[^ ^18^ ^]^ were proved to be effective in inducing cancer cells pyroptosis and triggering antitumor immunotherapy.^[^ ^19^ ^]^ However, clinical applications of chemotherapeutic drugs are restricted because of severe side effects and drug resistance.^[^ ^15^ ^]^ Therefore, it is vital to develop a more noninvasive and effective strategy to trigger pyroptosis for pancreatic cancer immunotherapy.

**Scheme 1:**
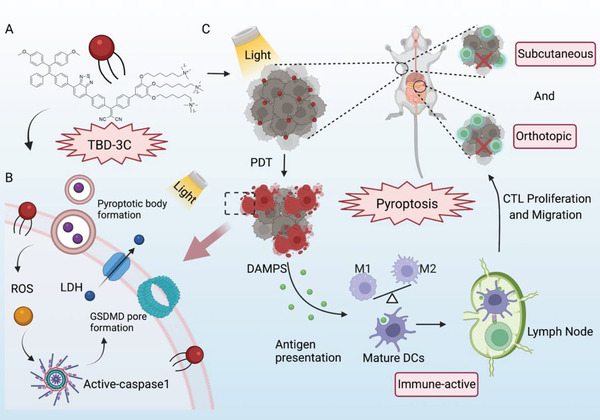
Schematic illustration of antitumor immunotherapy induced by photodynamic pyroptosis. A) Structure of membrane‐anchored photosensitizer named TBD‐3C, B) membrane anchoring TBD‐3C induces pyroptosis of tumor cells upon light irradiation, and C) further activates antitumor immunity and combats immunosuppressive microenvironment for cancer immunotherapy.

Unlike other treatment strategies such as ACT or ICIs which are responded to only a specific portion of patients, PDT has a higher patient response rate and is more flexible, controllable, and efficient. Over the past decade, PDT has been increasingly investigated and approved by regulatory agencies worldwide, with approved indications encompassing both skin lesions and solid tumors.^[^ ^20^, ^21^, ^22^ ^]^ PDT can induce apoptosis, autophagy, necrosis, and ICD, which is associated with various factors including the type of photosensitizer, light dose, and dose rate.^[^ ^22^ ^]^ In our previous research, we designed a novel membrane‐targeted photosensitizer named TBD‐3C with AIE feature which can activate cancer cell pyroptosis via PDT with noninvasiveness and mild side effects for the first time.^[^ ^23^ ^]^ Nevertheless, pyroptosis‐aroused cancer immunotherapies via PDT have rarely been reported. Herein, we evaluate whether TBD‐3C induced pyroptosis process could reverse the suppressive tumor microenvironment and arouse immunotherapy effect on pancreatic cancer both in vitro and in vivo. Our study demonstrates that a single effective light‐induced pyroptosis could activate antitumor immunity and contribute to a cascade of amplification effects in the tumor microenvironment (**Scheme** **1**). Such a strategy can significantly inhibit pancreatic cancer growth even for orthotopic xenograft tumors without additional treatment in the same model and set up a foundation for pyroptosis‐aroused immunotherapy of solid tumors.

## 2 Results and Discussion

### 2.1 TBD‐3C Induces Pancreatic Cancer Cell Death by Pyroptosis

**Figure 1:**
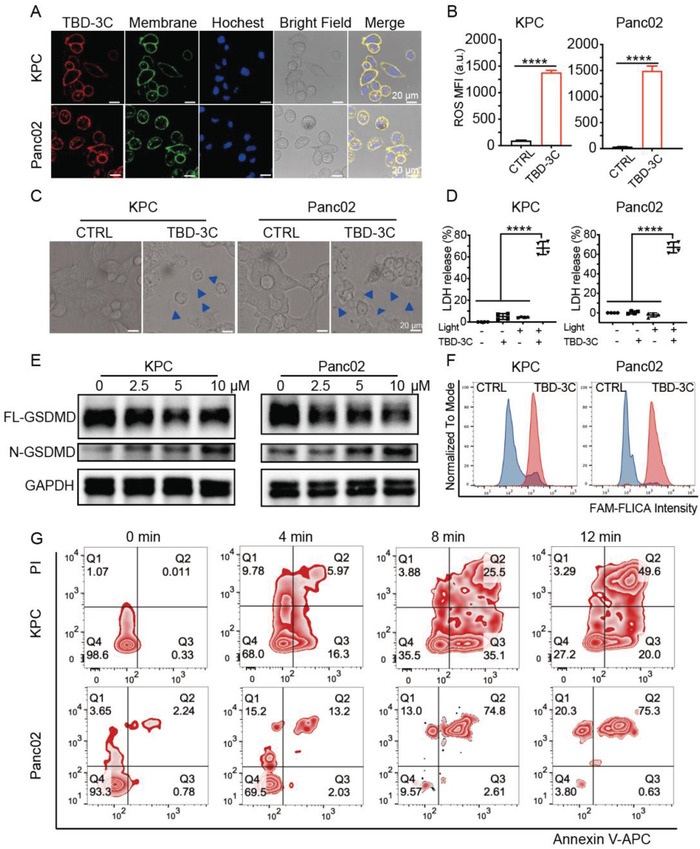
Membrane‐anchoring TBD‐3C stimulates the pyroptosis of pancreatic cancer cells by PDT. A) CLSM images of cells incubated with TBD‐3C (10 µm, 30 min, Red fluorescence, Ex is 405 nm, Em is from 600–680 nm), membrane tracker CellMask Green (Green fluorescence), and nuclei labeler (Blue fluorescence). Scale bar = 20 µm. B) ROS release of KPC and Panc02 cells labeled with TBD‐3C upon light irradiation at 40 mW cm^−2^ for 10 min (*n* = 3) (MFI, Mean Fluorescence Intensity). C) Confocal images of KPC and Panc02 cells labeled with TBD‐3C upon light irradiation at 40 mW cm^−2^ for 10 min, blue arrows indicate the cell membrane expansion. D) LDH generation of KPC and Panc02 cells labeled with TBD‐3C upon light irradiation at 40 mW cm^−2^ for 10 min (*n* = 4). E) Western blotting analysis of pyroptosis‐related protein expression (GSDMD‐FL, GSDMD‐N) in KPC cells and Panc02 cells after treatment with different concentrations of TBD‐3C after PDT stimulation. F) The caspase‐1 expression of KPC and Panc02 after PDT treatment by flow cytometry with FAM conjugated Caspase‐1 assay kit. G) Flow cytometry of propidium iodide and annexin V‐fluorescein isothiocyanate (APC)‐stained KPC and Panc02 cells labeled with TBD‐3C upon light irradiation at a power density of 40 mW cm^−2^ for 4, 8, 12 min, respectively. Data are presented as the mean ± SD. Statistical analysis was performed using the Student's *t*‐test (****p < 0.0001).

To investigate the feasibility of inducing pancreatic cancer pyroptosis by TBD‐3C under light irradiation, we selected two model murine cell lines KPC and Panc02, which were widely used in the study of pancreatic cancer. The red fluorescence from TBD‐3C in cancer cells was easily observed through a confocal laser scanning microscope (CLSM), where the cell membrane and nucleus were labeled with green membrane tracker and blue Hoechst 33342, respectively. TBD‐3C was mainly localized on the cell membrane as proved by the overlapped fluorescence of red and green (**Figure** **1**A). To investigate the optimal labeling concentration and staining duration of TBD‐3C on KPC and Panc02 cells, a quantitative analysis of labeling efficiency was performed by flow cytometry (Figure S1, Supporting Information). The results revealed that successful membrane anchoring of TBD‐3C on KPC and Panc02 cells could be achieved with the labeling time of 30 min at a concentration of 10 µm.

To further confirm the PDT effect of TBD‐3C in pancreatic cancer cell lines, the reactive oxygen species (ROS) generation capacity of TBD‐3C was additionally studied using flow cytometry with a cell‐permeable ROS sensor (DCFH‐DA) which sensitively emits fluorescence when encountered with ROS. As shown in Figure 1B, cells treated with TBD‐3C upon white light irradiation (40 mW cm^−2^) exhibited higher mean fluorescence intensity (MFI) than the control group, demonstrating increased ROS generation in KPC and Panc02 cells. Real‐time morphological tracking of the KPC and Panc02 cells after TBD‐3C treatment under white light was recorded by the lens of a microscope (Figure S2, Movies S1 and S2, Supporting Information). Cell swelling and large bulging bubbles on the plasma membrane were observed when TBD‐3C labeled KPC and Panc02 cells were irradiated for 10 min (40 mW cm^−2^) (Figure 1C), indicating a typical morphological characteristic of pyroptosis. To understand the pyroptotic effect of different light durations, we studied the images of KPC and Panc02 cells by CLSM after being treated with TBD‐3C (10 µm) upon light irradiation at a power of 40 mW cm^−2^ for 0, 4, 8, and 12 min (Figure S3, Supporting Information). These results showed that the degree of pyroptosis was higher with prolonging light irradiation.

Full‐length GSDMD (FL‐GSDMD) cleavage and lactate dehydrogenase (LDH) release are the typical features of pyroptotic cell death.^[^ ^24^ ^]^ Thus, GSDMD cleavage and LDH leakage from cells triggered by ROS were monitored. KPC and Panc02 cells treated with TBD‐3C upon white light irradiation (40 mW cm^−2^) showed a higher level of released LDH (Figure 1D) and N‐GSDMD (Figure 1E) than the control sample. Further, the caspase‐1 activity, which also plays a key role in pyroptosis, was detected with the FAM FLICA Caspase‐1 Assay Kit. This assay employed the fluorescent inhibitor probe FAM‐YVAD‐FMK to label active caspase‐1 enzyme in living cells in vitro. The result of flow cytometry (Figure 1F) showed that the fluorescence intensity in TBD‐3C treated group was much higher than the control group upon light irradiation, indicating that a higher level of caspase‐1 was produced by PDT treatment. These results collectively indicated that TBD‐3C could successfully activate the pyroptosis of pancreatic cancer cell lines.

Additionally, TBD‐3C labeled cells under irradiation were stained with propidium iodide (PI) and annexin V‐APC to analyze cell pyroptosis. As shown in Figure 1G, the percentage of double‐positive cells (pyroptotic cells) significantly increased with a longer irradiation time. When cells were stimulated by a well‐known apoptosis inducer staurosporine (STS), obvious apoptotic features including nuclear fragmentation (Figure S4, Supporting Information) and cleavage of poly ADP‐ribose polymerase (PARP) (Figure S5, Supporting Information) were observed. However, no obvious apoptotic feature was detected in TBD‐3C treated cells upon irradiation, suggesting that TBD‐3C treatment could barely induce apoptosis of KPC and Panc02 cells under irradiation. Next, the cell viability of the TBD‐3C was investigated by a cell counting kit‐8 (CCK‐8 kit) assay. KPC and Panc02 cells were cultured with TBD‐3C of different concentrations upon light illumination (40 mW cm^−2^) to investigate light‐triggered cell ablation. The result demonstrated that the IC50 values of TBD‐3C on KPC and Panc02 under illumination were 4 and 0.5 µm, respectively (Figure S6, Supporting Information). Negligible dark cytotoxicity of TBD‐3C was observed in both cell lines. The results implicated that the TBD‐3C treatment upon irradiation could efficiently induce KPC and Panc02 cells’ death by pyroptosis.

### 2.2 Photodynamic Pyroptosis Promotes Immune Cells Activation In Vitro

**Scheme 2:**
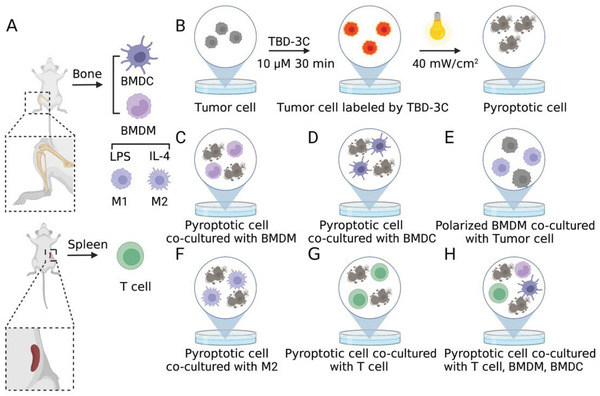
Schematic illustration of the co‐incubation system to simulate tumor immune microenvironment. A) Schemes showing the isolation of BMDC, BMDM, T lymphocytes, and the polarity of M1 and M2. B) The tumor cells were pre‐treated with TBD‐3C upon light illumination to obtain pyroptotic cells before co‐culture. C) Pyroptotic cells were co‐cultured with BMDM. D) Pyroptotic cells were co‐cultured with BMDC. E) The polarized BMDM cells were collected to co‐culture with fresh tumor cells again. F) Pyroptotic cells were co‐cultured with M2. G) Pyroptotic cells were co‐cultured with T lymphocytes. H) Pyroptotic cells were co‐cultured with T lymphocytes, BMDM, and BMDC.

Several studies have proved that tumor cell pyroptosis ultimately triggers ICD and can remarkably boost antitumor immunity.^[^ ^11^ ^]^ In this part, we performed a series of experiments to investigate whether PDT‐arousing pyroptosis by TBD‐3C could trigger ICD and initiate antitumor immunity. As a typical feature, DAMPs such as calreticulin (CRT) were released during the process of ICD. CRT shifted to the cell membrane to facilitate APC recruitment, recognition, and antigen presentation and initiated the immune response.^[^ ^25^ ^]^ Therefore, we used an anti‐CRT antibody to examine CRT exposure on KPC and Panc02 cells. The quantification results (Figure S7, Supporting Information) confirmed that the KPC and Panc02 cells treated with TBD‐3C under irradiation significantly enhanced the expression of CRT on the cell surface. DC and macrophages are both important innate immune cells, which play crucial roles in phagocytic clearance, antigen presentation, and initiating adaptive antitumor immunity.^[^ ^26^ ^]^ Typically, macrophages can be classified as M1 or M2 macrophages.^[^ ^27^ ^]^ The M1 subtype is the strong killer of cancer cells, while the M2 subtype is beneficial to sustaining immune suppressiveness.^[^ ^28^, ^29^ ^]^ Moreover, it is well established that CTLs‐based immunity is the major contributor to cancer immunotherapy. Thus, these three kinds of immune cells were chosen to represent the TME of pancreatic cancer in vitro. Subsequently, a scientific co‐incubation system containing pyroptotic cells induced by TBD‐3C co‐cultured with mice bone marrow‐derived dendritic cells (BMDCs), mice bone marrow‐derived macrophages (BMDMs), and mice spleen‐derived T lymphocytes was designed to mimic the natural TME (detailed steps in **Scheme** **2**). The purity of BMDCs, BMDMs (M0), and T lymphocytes isolated from mice and the polarity of M1 and M2 macrophages were confirmed by flow cytometry and shown in Figure S8 (Supporting Information).

**Figure 2:**
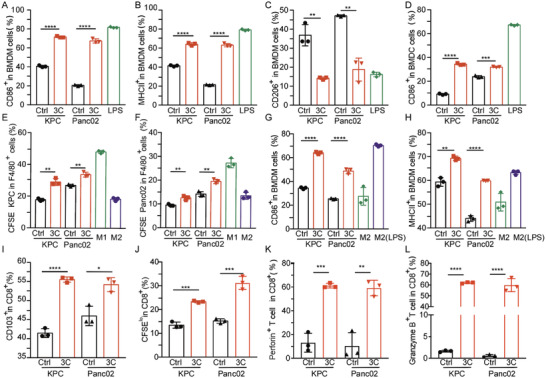
In vitro immune activation by PDT‐aroused pyroptosis. The quantification of A) CD86^+^ M1, B) MHC II^+^ M1, and C) CD206^+^ M2 after co‐culture in different groups. D) The quantification of mature DC cells after co‐culture in various groups. E) The quantification of CFSE‐KPC cells in the BMDM cells. F) The quantification of CFSE‐Panc02 cells in the BMDM cells. The quantification of G) CD86^+^ M1 and H) MHC II^+^ M1 after co‐culture. Assessment of I) mature T cell marker (CD103) and J) the marker for proliferated T cells (CFSE). Assessment of K) perforin and L) granzyme B markers on CD8^+^ T cells after co‐culture with BMDM and BMDC cells. Data are presented as the mean ± SD. Statistical analysis was performed by Student's *t*‐test (*p < 0.05, **p < 0.01, ***p < 0.001, and ****p < 0.0001) (*n* = 3).

Firstly, we examined whether the treatment of pyroptotic cells could induce the M1‐polarization of macrophages and the maturation of DCs. BMDMs and BMDCs were isolated from the bone marrow of mice in advance (Scheme 2A). KPC and Panc02 cells treated by TBD‐3C (10 µm) upon light illumination (40 mW cm^−2^) (Scheme 2B) were collected as pyroptotic cells, followed by separate co‐culturing with BMDMs (Scheme 2C) and BMDCs (Scheme 2D) in the plates at the ratio 1:1 (1 × 10^5^) for 48 h to evaluate the phenotype transformation of BMDMs and BMDCs. Lipopolysaccharide (LPS) can polarize macrophages and activate the immune response. Thus, treatments with LPS were conducted as a positive reference in the following experiments.^[^ ^30^, ^31^ ^]^ M1‐like macrophage, M2‐like macrophage, and mature DC were defined by CD86^hi^ MHC II^hi^ CD206^lo^, CD86^lo^ MHC II^lo^ CD206^hi^, and CD86^hi^, respectively,^[^ ^30^, ^32^ ^]^ so that the related markers were detected by flow cytometry. The expression of CD86 and MHC II on the BMDMs as well as the CD86 expression level on the BMDCs were 2–3 folds higher than the negative control plate and had the increasing tendency as the LPS stimulated group (**Figure** **2**A,B,D). In addition, we also detected the CD206 expression on the BMDMs (Figure 2C) after treatment with pyroptotic cancer cells, which showed lower expression levels than that of the control group and comparable expression levels to LPS stimulated group. Their representative cytometry patterns are shown in Figures S9 and S10 (Supporting Information). The co‐culture assay demonstrated that the pyroptotic cancer cells exhibited outstanding efficacy in stimulating M1 phenotype macrophage differentiation and BMDC maturation.

Phagocytic clearance is also an important indicator to evaluate the ability of BMDM to suppress tumor growth.^[^ ^33^ ^]^ To better observe phagocytic clearance of pyroptotic tumor cells polarized BMDM toward fresh cancer cells, the fresh cancer cells including KPC and Panc02 were pre‐labeled with carboxyfluorescein succinimidyl ester (CFSE), namely CFSE‐KPC and CFSE‐Panc02. Then, CFSE‐KPC and CFSE‐Panc02 were separately cultured with the pyroptotic tumor cells polarized BMDM (F4/80^+^) at the ratio of 1:1 for 4 h (Scheme 2E). LPS‐induced M1‐like and IL‐4‐induced M2‐like macrophages treated with CFSE‐KPC or CFSE‐Panc02 were respectively set as the positive control and negative control for phagocytic clearance of cancer cells. The percentage of BMDM (F4/80^+^) cells that had engulfed CFSE‐KPC or CFSE‐Panc02 was measured by flow cytometry. As shown in Figure 2E,F, the percentages of both BMDM (F4/80^+^) containing CFSE‐KPC and BMDM (F4/80^+^) containing CFSE‐Panc02 had significantly increased after being treated with pyroptotic tumor cells polarized BMDM (F4/80^+^). These results indicated that the macrophages exposed to pyroptotic tumor cells could enhance their phagocytic ability toward tumor cells, which would contribute to further tumor ablation.

TAMs are potentiated to more closely M2‐polarized macrophages to inhibit antitumor immune response and even promote tumor progression.^[^ ^34^ ^]^ To further explore whether pyroptotic tumor cells could re‐educate the existing M2 to polarize to M1 in the TME, IL‐4‐induced M2‐like macrophages were co‐cultured with pyroptotic tumor cells for 48 h, then the expression of CD86 and MHC II on the macrophages were detected again (Scheme 2F). As shown in Figure 2G,H, the expression of CD86 and MHC II were significantly increased as compared to that of the negative control plate, which was close to the LPS positive control group. Together, these results indicated the existing M2 could be re‐educated toward M1 to inhibit the tumor growth by PDT‐induced pyroptotic tumor cells.

The impact of pyroptotic tumor cells on T lymphocytes was studied subsequently. The T lymphocytes were co‐incubated with the pyroptotic tumor cells, BMDMs, and BMDCs for 48 h (Scheme 2H). We concentrated on the CFSE‐labeled CD8^+^ T cells which are recognized as the heart of adaptive immunity. CD103 and CFSE levels representing the maturation and proliferation of CD8^+^ T cells were then detected. Compared with the untreated group, the PDT‐arousing pyroptotic cells significantly promoted CD8^+^ T cell proliferation and maturation with the existence of BMDMs and BMDCs (Figure 2I,J). The representative cytometry patterns are shown in Figure S11 (Supporting Information). Granzyme B and perforin are responsible for the CTLs‐based immunity. Activated CD8^+^ CTLs can secrete granzyme B and perforin to lyse the target cell and cause its death by triggering apoptosis or other forms of cell death.^[^ ^35^ ^]^ Thus the in vitro CD8^+^ T cell activation was further evaluated by analyzing granzyme B and perforin secretion via flow cytometry. T lymphocytes were co‐incubated with pyroptotic tumor cells with or without BMDMs and BMDCs (Scheme 2G,H). PDT‐arousing pyroptotic cells by TBD‐3C significantly stimulated CD8^+^ T cells to secrete perforin and granzyme B with the existence of BMDMs and BMDCs, where the percentage of perforin^+^ CD8^+^ T cells and granzyme B^+^ CD8^+^ T cells were respectively sixfold and 60‐fold of control groups (Figure 2K,L and Figure S12, Supporting Information). The representative cytometry patterns are shown in Figure S13 (Supporting Information). These results jointly revealed the great potential of TBD‐3C PDT treatment for both BMDM‐to‐T and DC‐to‐T immune activation, indicating that PDT‐arousing tumor pyroptosis by TBD‐3C could enable the co‐delivery of tumor antigens and adjuvant with high immunogenicity to cause antitumor immune response.

### 2.3 Pyroptosis Remodeling Tumor Microenvironment to Promote Antitumor Immunotherapy

**Figure 3:**
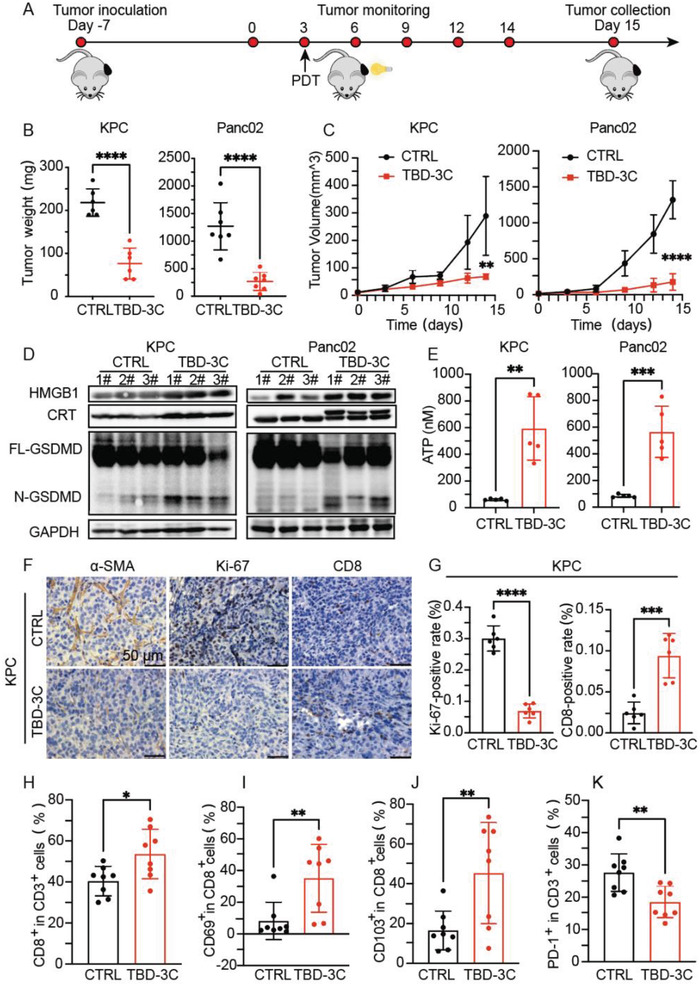
In vivo antitumor effects of localized delivery of TBD‐3C in KPC and Panc02 tumor‐bearing mice. A) Schematic illustration of TBD‐3C‐based PDT‐arousing pyroptosis to inhibit tumor growth. C57 mice were subcutaneously inoculated with 5 × 10^5^ KPC cells or 2 × 10^5^ Panc02 cells on day 7. PBS or TBD‐3C was then intratumorally injected on day 3 and under white light irradiation (40 mW cm^−2^, 10 min) for 24 h after injection. B) The tumor weights and C) tumor growth curves of various treatment groups. D) Western blotting analysis of HMGB1, CRT, GSDMD‐FL, and GSDMD‐N in tumor tissue after treatments (*n* = 3). E) Quantitative analysis of ATP in tumor tissue after treatments (*n* = 5). F) Pathology studies show *α*‐SMA, Ki‐67, and CD8 expression in the mice in different groups in the KPC model. The bars represent 50 µm. G) Ki‐67 and CD8 positivity analyses in the KPC model (*n* = 5). Flow cytometry quantification of H) CD8, I) CD69, J) CD103, and K) PD‐1 expression on intratumoral infiltration CTLs with TBD‐3C photodynamic treatment in the KPC model. Data are presented as the mean ± SD. Statistical analysis was performed by Student's *t*‐test (*p < 0.05, **p < 0.01, ***p < 0.001, and ****p < 0.0001).

The successful immune activation of PDT‐aroused pyroptosis by TBD‐3C in vitro drove the subsequent hypothesis that PDT‐aroused pyroptosis could suppress tumor growth and remodel TME. Subsequently, we evaluated the inhibition efficacy of TBD‐3C treatment under irradiation on the progression of pancreatic cancer. Subcutaneous pancreatic cancer (KPC and Panc02) models in immunocompetent C57 mice were established. TBD‐3C dissolved in PBS or PBS was intratumorally injected followed by light irradiation (40 mW cm^−2^, 10 min) when the average tumor volume reached 50 mm^3^ (**Figure** **3**A). At the end of treatment, the main organs and tumors from all sacrificed mice were collected for further detection. The tumor weights (Figure 3B) and tumor volume growth curves (Figure 3C and Figure S14A–D, Supporting Information) showed that PDT with TBD‐3C has significantly inhibited tumor growth as compared with the PBS group. The corresponding excised tumor images after treatment are shown in Figure S14E,F (Supporting Information). No body weight loss occurred, indicating negligible side effects of TBD‐3C PDT treatment (Figures S14G and S15H, Supporting Information). Besides, mice that received TBD‐3C PDT treatment exhibited longer survival compared with the mice treated with PBS (Figure S15, Supporting Information). Without irradiation, treatment with TBD‐3C showed no inhibitory effect on tumor progression (Figure S16, Supporting Information). To further confirm that TBD‐3C could induce pyroptosis in vivo via PDT, we examined the LDH release and GSDMD cleavage in tumors. The TBD‐3C PDT group showed a higher level of released LDH (twofold) (Figure S17, Supporting Information) and more FL‐GSDMD was cleaved (Figure 3D) than the control group both in KPC and Panc02 model. To further prove the pyroptosis‐mediated ICD by TBD‐3C PDT in vivo, the TAAs marker CRT and high mobility group box 1 (HMGB1) in tumor tissue were also investigated via Western blot and immunofluorescence. The results in Figure 3D and Figure S18 (Supporting Information) showed that the TBD‐3C PDT group exhibited higher expression of HMGB1 and CRT both in KPC and Panc02 model. Moreover, adenosine triphosphate (ATP) release as an important ICD marker was also monitored, the results in Figure 3E showed tumors treated with TBD‐3C PDT exhibited increased ATP secretion. Immunohistochemistry staining of tumor sections was performed with anti‐Ki‐67 antibody for assessing cell proliferation, anti‐*α*‐SMA antibody for fibrosis marking, and anti‐CD8 antibody for CD8^+^ CTL infiltration. The results revealed that TBD‐3C photodynamic treatment had lowered Ki‐67 positive rate, reduced *α*‐SMA expression, and increased CD8^+^ CTL rate in comparison with the control group (Figure 3F, Figure S19A, Supporting Information). Corresponding quantitative analysis is shown in Figure 3G and Figure S20B (Supporting Information). In addition, TBD‐3C photodynamic treatment showed no hemocytolysis effect in blood circulation and non‐toxicity to organs (Figure S20, Supporting Information). The blood biochemistry exhibited no abnormality after TBD‐3C photodynamic treatment when compared to the PBS control group (Figure S21, Supporting Information). These results suggest the excellent antitumor effects upon light irradiation and good biocompatibility of TBD‐3C PDT.

CD8^+^ CTLs play a dominant role in the antitumor immune response by enhancing tumor cell elimination after being activated by APCs presenting tumor antigens.^[^ ^36^ ^]^ We evaluated the ability of photodynamic pyroptosis by TBD‐3C to promote the activation of CD8^+^ CTLs. As shown in Figure 3H, the percentage of tumor‐infiltrating CTLs after TBD‐3C photodynamic treatment was 53.64 ± 12.1%, which was higher than the control group (40.46 ± 7.2%). Upregulation of CD69 expression is a trait of CTLs activation and the percentage of CD69^+^ T cells in CD8^+^ T cells was markedly increased in the tumor after being treated with PDT, which is almost 18‐fold higher than that in the control group (Figure 3I). Besides, TBD‐3C photodynamic treatment also induced CD103 upregulation on CD8^+^ T cells (Figure 3J), which plays an essential role in inhibiting tumor growth. Programmed death 1 (PD‐1) is an immune inhibitory receptor, and PD‐1 overexpression on CTLs is elevated with tumor immune evasion.^[^ ^37^ ^]^ Thus, the PD‐1 expression on CTLs cells was evaluated as shown in Figure 3K. We found that the expression of PD‐1 on CTLs (18.53 ± 4.9%) with the TBD‐3C photodynamic treatment was significantly decreased than that of control groups (27.64 ± 5.8%). Downregulating of PD‐1 on CTLs could evade immune checkpoint identification and would further contribute to the inhibition of tumor immune escape. The representative cytometry patterns are shown in Figure S22 (Supporting Information). The cytokines in the serum such as tumor necrosis factor‐*α* (TNF‐*α*) and interferon‐*γ* (IFN‐*γ*) are closely associated with antitumor immunity. Mice treated with TBD‐3C PDT exhibited significantly elevated concentrations of TNF‐*α* and IFN‐*γ* in serum (Figure S23, Supporting Information) compared to those treated with PBS, indicating that TBD‐3C PDT treatment caused a stronger antitumor immune response.

**Figure 4:**
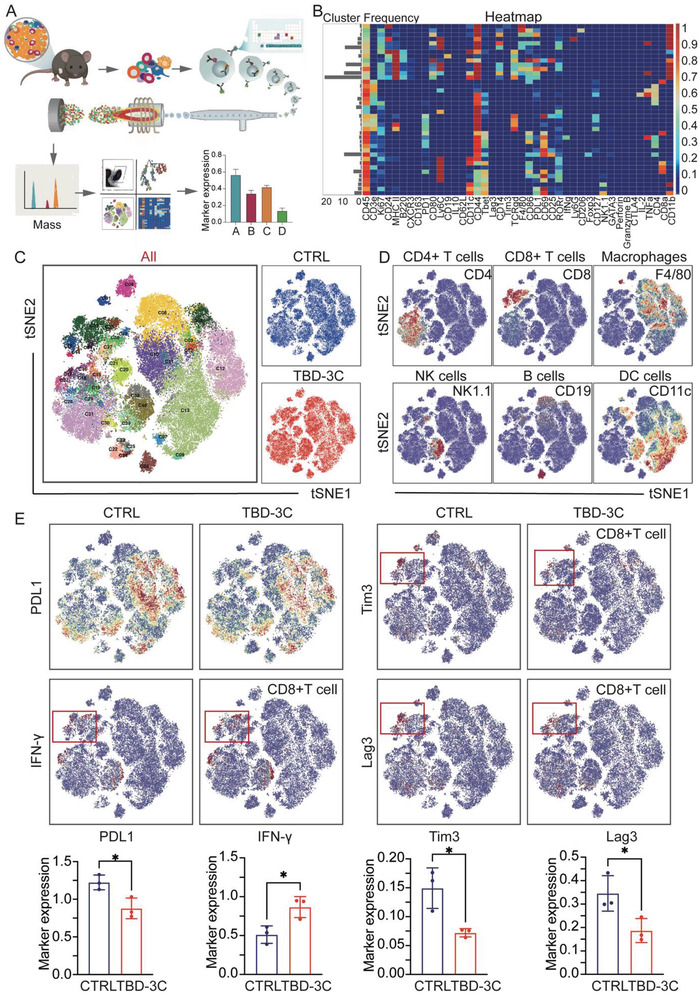
Mass cytometry analysis of lymphocytes in TME after photodynamic pyroptosis therapy in KPC‐bearing mice. A) A brief scheme demonstrating the sample processing and data analysis process. B) A heat map showing the normalized expression of the markers. C) A *t*‐SNE map was colored by 40 clusters from all samples and the blue one represented the control group, the red one represented the TBD‐3C PDT group. D) Normalized expression of markers for the six main immune cell types on the *t*‐SNE map. E) *T*‐SNE plots showing the expression of marker PD‐L1, IFN‐*γ*, Tim3, and Lag3 in different groups and relevant statistics are shown together. Data are presented as the mean ± SD. Statistical analysis was performed by Student's *t*‐test (*p < 0.05 and**p < 0.01) (*n* = 3).

To understand the transformation of the immune landscape in TME that was affected by the TBD‐3C aroused pyroptosis via PDT, we conducted a high‐dimensional characterization of KPC‐bearing mice tumors from the two groups by time‐of‐flight mass cytometry (CyTOF), which could concurrently detect abundant biomarkers expression and comprehensive immunological information (**Figure** **4**A). The heatmap (Figure 4B) and two‐dimensional *t*‐stochastic neighbor embedding (*t*‐SNE) projections (Figure 4C) were constructed for visualization, where the immune cells were divided into 40 clusters by 42 immune markers. The excel containing the detailed definition of each cluster based on the different levels of marker expression could be obtained from Table S2 (Supporting Information). Representative images of cell proportion in different groups are also shown in Figure S24 (Supporting Information). Overall, the immune landscape in time‐of‐flight mass cytometry was changed in the TBD‐3C PDT group as compared with the control group (Figure 4C and Figure S25, Supporting Information). For convenience, six main kinds of cell types including CD4^+^ T cells, CD8^+^ T cells, macrophages, natural killer (NK) cells, B cells, and DC cells were identified among the clusters based on typically expressed markers (Figure 4D). Especially, the overall expression of PD‐L1 and the markers including lymphocyte activation gene‐3 (Lag3) and T cell immunoglobulin‐3 (Tim3) on CD8^+^ T cells (Cluster 35) in the TBD‐3C PDT group were downregulated significantly, and all of which are immune suppressive markers. Excitingly, the expression of IFN‐*γ* (Interferon‐gamma) on CD8^+^ T cells was increased observably (Figure 4E). Moreover, the numbers of immune‐active cells, such as *γδ* T cells, NK cells, and CD4^+^ TEMRA (effector memory CD45RA^+^) (Figure 4F), were increased remarkably in the treatment group as compared with those in the control group. Besides, the immunosuppressive myeloid‐derived suppressor cells (MDSCs) were also reduced significantly in the TBD‐3C PDT group (Figure S26, Supporting Information). Collectively, these results demonstrated that the PDT‐aroused pyroptosis by TBD‐3C successfully converted the immune landscape from immunosuppressive “cold” TME to immunogenic “hot” TME.

### 2.4 Pyroptosis‐Aroused Immunological Responses to Attack Distant Orthotopic Tumor

**Figure 5:**
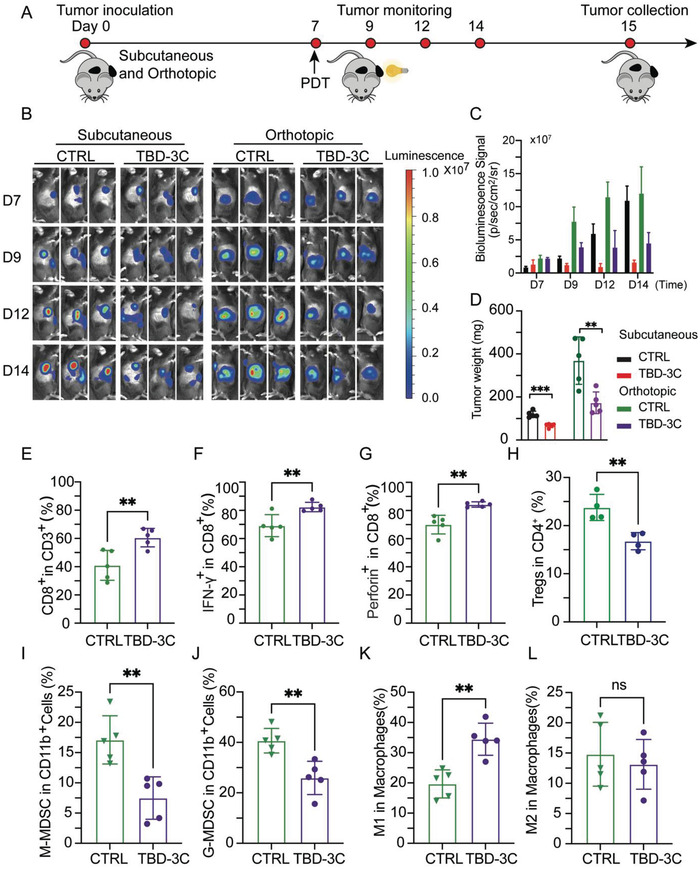
System immunity activated by PDT‐arousing pyroptosis. A) Schematic illustration of PDT‐arousing pyroptosis by TBD‐3C to a distant orthotopic tumor. C57 mice were subcutaneously and orthotopically inoculated with KPC cells on day 0. PBS or TBD‐3C was then intratumorally injected (subcutaneous) on day 7 and under white light irradiation (40 mW cm^−2^, 10 min) 24 h later. B) In vivo bioluminescence imaging of the KPC tumors (both subcutaneous and orthotopic) in control and treated groups. Three representative mice of each group are shown. C) The quantitative bioluminescence signals of tumors in the different groups at different time points. D) The weights of subcutaneous and orthotopic tumors in different groups. Flow cytometry quantification of E) CD8^+^ T cells, F) IFN‐*γ* ^+^CD8^+^ T cells, G) perforin^+^CD8^+^ T cells, and H) Tregs in the excised tumors collected from KPC orthotopic xenograft model. Statistic results for the proportions of I) M‐MDSC, J) G‐MDSC, K) M1 macrophages, and L) M2 macrophages in the TME of KPC orthotopic xenograft model. Data are presented as the mean ± SD. Statistical analysis was performed by Students’ *t*‐test (ns, nonsignificant. **p < 0.01 and ***p < 0.001).

After confirming that the TBD‐3C aroused pyroptosis could suppress tumor growth and remodel the TME of the “local tumor,” we further assessed whether the local TBD‐3C PDT treatment could induce anti‐tumor immune responses to treat “distant tumor” effectively. C57BL/6 male mice were subcutaneously inoculated with 5 × 10^5^ KPC‐Luc cells at the right flank to build a subcutaneous tumor model (local tumor) and injected with 5 × 10^5^ KPC‐Luc cells at the tail of the pancreas to build an orthotopic tumor model (distant tumor) in every mouse. As depicted in **Figure** **5**A, mice were randomly divided into two groups with the same density of bioluminescence signals at 7 d post‐tumor inoculation, which were treated with PBS or TBD‐3C via intratumoral injection (local tumor). After 24 h injection, mice were treated with white light irradiation (40 mW cm^−2^, 10 min). To evaluate the therapeutic efficacy of TBD‐3C PDT treatment in both subcutaneous and orthotopic models, the tumor growth was monitored using the In Vivo Imaging System (IVIS). The tumor size was measured on day 7, 9, 12, and 14. Bioluminescence images and corresponding bioluminescence signals of subcutaneous and orthotopic tumors are shown in Figure 5B,C. It was found that the mice receiving TBD‐3C PDT treatment showed remarkable inhibition of both subcutaneous tumor and orthotopic tumor growth, suggesting that our strategy could promote effective host antitumor immune responses. Consistently, the images (Figure S27, Supporting Information) and weights (Figure 5D) of excised subcutaneous and orthotopic tumors from KPC‐bearing mice at the end of treatment showed that the tumor size of the treatment group was much smaller than those in the control group. Next, we performed flow cytometry to quantitate the naïve T cells (CD44^low^CD62L^hi^), central memory T cells (T_CM_) (CD44^hi^CD62L^hi^), and effector memory T cells (T_EM_) (CD44^hi^CD62L^low^) in the spleen which is a crucial characterization for distal tumor inhibition (Figure S28A, Supporting Information). The T_EM_ in the TBD‐3C PDT group was significantly more than that in the PBS group, while the proportion of naïve and T_CM_ indicated no difference in different groups (Figure S28B–D, Supporting Information). These results indicate that mice treated with TBD‐3C PDT generate an immune memory effect to inhibit distant tumors. To provide a clear illustration of immune reaction in the distant tumor, we utilized flow cytometry to quantitate CD8^+^ T cells, IFN‐*γ* ^+^CD8^+^ T cells, perforin^+^CD8^+^ T cells, and regulatory T cells (Tregs) in the orthotopic tumor. The TME analysis showed that the TBD‐3C PDT treatment group had promoted the infiltration of CD8^+^ T cells, IFN‐*γ* ^+^CD8^+^ T cells, and perforin^+^CD8^+^ T cells in tumors while decreasing the infiltration of Tregs compared to that in the tumors treated with the control group (Figure 5E–H; Figure S29A, Supporting Information). Furthermore, the TME analysis of orthotopic tumors showed that the TBD‐3C PDT treatment group could significantly decrease the percentage of Monocytic‐MDSCs (M‐MDSCs) (CD45^+^CD11b^+^Ly6C^+^Ly6G^−^) and Granulocytic‐MDSCs (G‐MDSCs) (CD45^+^CD11b^+^Ly6C^−^Ly6G^+^) in the CD11b^+^CD45^+^ cells, which are immunosuppressive in TME. (Figure 5I,J; Figure S29B, Supporting Information). The changes of M1 and M2 subtypes were also analyzed respectively. As shown in Figure 5K,L, the proportion of M1 macrophages was significantly increased in the TBD‐3C PDT group compared with the PBS group while there was no significant change in the portion of M2 macrophages. Collectively, these results provided a clear illustration of immune activation in the distant tumor and imply the immunotherapeutic activity in the TME of distant tumors, suggesting the great potential of TBD‐3C PDT for the treatment of tumor metastasis.

## 3 Conclusions

In conclusion, we report a strategy for immunotherapy through cell pyroptosis by a membrane‐targeted AIE photosensitizer (TBD‐3C), which demonstrated an excellent antitumor efficiency for pancreatic cancer. TBD‐3C showed high tumor cell membrane labeling efficiency, and precise light‐controlled cell pyroptosis activating ability which led to TME remodeling with strong APC maturation and T lymphocyte infiltration, and high efficiency in tumor growth suppression both in subcutaneous and orthotopic xenograft models. This study provides a foundational platform for light‐controlled antitumor immunity aroused by cell pyroptosis with high biocompatibility, opening a window on tumor immunotherapy.

## 4 Experimental Section

### Ethics Statement

The manuscript was written according to established ethical standards. The experimental protocol was approved by the ethical standards of the Ethics Committee of the First Affiliated Hospital of Zhejiang University School of Medicine. The animal experiments were performed following the applicable guidelines of the Animal Ethics Committee of the First Affiliated Hospital of Zhejiang University School of Medicine and the China Animal Protection Law.

### Materials

TBD‐3C used in this study was prepared according to the previous research.^[^ ^23^ ^]^ The other agents used in this study were as following: McCoy's 5A (Modified) Medium (Thermo Fisher Scientific, 16600082), RPMI 1640 Medium (Gibco, 21870076), Fetal bovine serum (FBS) (Gibco, 10100147), Hoechst 33342 (Solarbio, C0031), Membrane tracker CellMask Green (Thermo Fisher Scientific, C37608), Reactive Oxygen Species Assay Kit (Beyotime, C0033), LDH Cytotoxicity Assay Kit (Beyotime, C0016), Annexin V‐PI Apoptosis Detection Kit (Elabscience, E‐CK‐A217), Cell Counting Kit‐8 (Beyotime, C0037), Staurosporine (STS) (MCE, 15141), BCA assay (Beyotime, P0012), ATP assay kit (Beyotime, S0026), GAPDH antibody (Beyotime, AF5009), PRAP antibody (Cell Signaling Technology, 9542), GSDMD antibody (Cell Signaling Technology, 96458), Alexa Fluor 647 Anti‐Calreticulin (Abcam, ab196159), FAM‐FLICA Caspase‐1 (YVAD) Assay Kit (Immunochemistry, 97), Red blood cell lysis buffer (Beyotime, C3702), Mouse IL‐4 (Peprotech, 214‐14), Mouse M‐CSF (Peprotech, 315‐02), Mouse GM‐CSF (Peprotech, 315‐03), LPS (Sigma‐Aldrich, L5293), CD8^+^ T cell isolation kit (Miltenyibiotec, 130‐096‐495), Dispase (Gibco, 17105041), Collagenase IV (Thermo Fisher Scientific, 17104019), Calcium chloride solution (Sigma‐Aldrich, 21115), DNase (Sigma‐Aldrich, D5025), Percoll (GE Healthcare, 17‐0891‐01), anti‐mouse CD16/32 antibody (Biolegend, 101320), Fixation/Permeabilization solution kit (BD Biosciences, 555028), Leukocyte activation cocktail (BD Biosciences, 550583), anti‐CD45‐BV605 (BD Biosciences, 563053), anti‐CD45‐BV786 (BD Biosciences, 564225), anti‐CD3‐FITC (BD Biosciences, 555274), anti‐CD4‐APC‐Cy7 (BD Biosciences, 552051), anti‐CD8‐PE‐Cy7 (BD Biosciences, 552877), anti‐CD86‐PE (BD Biosciences, 553692), anti‐MHC II‐BV421 (BD Biosciences, 562564), anti‐CD103‐AF700 (BD Biosciences, 565529), anti‐CD206‐AF647 (BD Biosciences, 565250), anti‐CD11b‐PE‐CF594 (BD BioSciences, 562399), anti‐CD11c‐PC5.5 (BD BioSciences, 560584), anti‐IFN‐*γ*‐APC (BD Biosciences, 554413), anti‐PD‐1‐BV421 (BD Biosciences, 562584), anti‐CD49b‐APC (BD Biosciences, 558295), anti‐CD69‐BV786 (BD Biosciences, 564683), anti‐F4/80‐PE‐Cy7 (Biolegend, 157308), anti‐Granzyme B‐PC5.5 (BioLegend, 396412), anti‐Perforin‐PE (BioLegend, 154306), anti‐CD25‐AF700 (Biolegend, 102024), DAB Chromogen kit (Biocare, BDB2004), Rabbit anti‐CD8 (Cell Signaling Technology, 98941), Ki‐67 antibody (Cell Signaling Technology, 10020), *α*‐SMA antibody (Cell Signaling Technology, 19245), HMGB1 antibody (Servicebio, GB11103), Calreticulin (CRT) antibody (Servicebio, GB112134), anti‐CD44‐BV510 (BD Biosciences, 563114), anti‐CD62L‐PE (BioLegend, 104408), anti‐Foxp3‐PE (BioLegend, 126403), anti‐Ly6C‐BV605 (BD Biosciences, 563011), anti‐Ly6G‐FITC (BD Biosciences, 551460), Mouse IFN‐*γ* Precoated Elisa Kit (DaYou, 1210002), Mouse TNF‐*α* Precoated Elisa Kit (DaYou, 1217202), LIVE/DEADTM Fixable Violet Dead Cell Stain Kit (Thermo Fisher Scientific, L34963).

### Confocal Imaging

To prove the membrane anchoring ability of TBD‐3C, the KPC and Panc02 cells were seeded in an 8‐well chamber and grown to 70% density before assay. The cells were incubated with fresh cell culture medium containing TBD‐3C (10 µm) for 30 min. After incubation, the cells were stained by membrane tracker CellMask Green (2 µg mL^−1^) for 15 min after twice washing with PBS. At last, the cell nuclei were stained by Hoechst 33342 (10 µg mL^−1^) for 5 min before cell imaging. TBD‐3C channel: Ex = 405 nm, Em = 600–680 nm; Green CellMask channel: Ex = 522 nm, Em = 530–540 nm; Blue Hoechst 33342 channel: Ex = 405 nm, Em = 430–470 nm. To capture the cell morphology change, the KPC and Panc02 cells were cultured with culture medium containing TBD‐3C (10 µm) for 30 min and then upon light irradiation at 40 mW cm^−2^ for 10 min. After 1 h culture, the cells were monitored by bright‐field images with CLSM. Real‐time morphological tracking of the KPC and Panc02 cells after TBD‐3C (10 µm) treatment under white light (405 nm, 8%) was recorded by LEICA SP8 confocal microscope.

### ROS Assay

The DCFH‐DA was utilized as a ROS indicator to detect ROS generation of TBD‐3C in KPC and Panc02 cells. KPC and Panc02 cells were seeded in a 6‐well plate (1 × 10^5^ cells each well) and cultured overnight. Afterward, cells were incubated with a fresh medium containing TBD‐3C (10 µm) for 30 min followed by irradiation at 40 mW cm^−2^ (10 min). The cells treated with PBS were set as control. After 24 h culture, the cells were washed with PBS followed by DCFH‐DA (10 µm) staining at 37 °C for 15 min. The fluorescence intensity was detected by flow cytometry with excitation at 488 nm.

### LDH Release Assay

KPC and Panc02 cells were seeded in 96‐well plates (1 × 10^4^ per well) and incubated overnight. The next day, the cells were treated by PBS or TBD‐3C (10 µm) upon irradiation at 40 mW cm^−2^ or under dark. After 24 h incubation, the activity of LDH released into cell culture supernatants was monitored by the LDH Cytotoxicity Assay Kit according to the manufacturer's instructions.

### In Vitro Annexin V‐FITC/PI Assay

KPC and Panc02 cells were seeded into 6‐well plates at a density of 1 × 10^5^ per well and incubated overnight. Then, cells were incubated with TBD‐3C (10 µm) for 30 min and followed by light irradiation at 40 mW cm^−2^ for 0, 4, 8 and 12 min. After 24 h incubation, the cells were collected and stained with Annexin V‐APC/propidium iodide (PI) according to the instructions. The cells were detected by flow cytometry using a LSRFFortessa Cell Analyzer (BD Biosciences) and data were analyzed by FlowJo software.

### In Vitro Co‐Culture Assay

Briefly, the co‐culture system consists of pyroptotic cells induced by TBD‐3C, BMDCs, mice BMDMs, and mice spleen‐derived T lymphocytes. Next, each part of the experiment was specified. To obtain the pyroptotic cells, KPC and Panc02 cells were seeded into 6‐well plates at a density of 1 × 10^5^ per well and incubated overnight. Then, cells were incubated with TBD‐3C (10 µm) for 30 min and followed by light irradiation at 40 mW cm^−2^ for 10 min. The KPC and Panc02 cells treated with PBS were set as control. To analyze the macrophage polarization, the isolated BMDMs were co‐cultured with pyroptotic cells or control cells at the ratio of 1:1. After 48 h incubation, the cells in the plate were collected and analyzed by flow cytometry. The cells were incubated with CD11b, CD86, MHC II, and CD206 antibodies. The CD11b antibody was used to gate the BMDM group for further analysis. The BMDMs treated with LPS (0.5 µg mL^−1^) for 48 h were set as a positive reference. To analyze the DC maturation, the isolated BMDCs were co‐cultured with pyroptotic cells or control cells at the ratio 1:1 for 48 h. Then, the cells in the plate were collected and analyzed by flow cytometry. The cells were incubated with CD11b and CD86 antibodies and the CD11b antibody was used to gate the BMDC group. The BMDCs treated with LPS (0.5 µg mL^−1^) for 48 h were set as positive reference. To analyze the phagocytic clearance of pyroptotic tumor cells polarized BMDM, the polarized BMDMs were collected after 48 h incubation. The KPC and Panc02 cells were labeled with 4 µmol L^−1^ CFSE for 10 min at 37 °C, namely CFSE‐KPC and CFSE‐Panc02. Then, the CFSE‐KPC and CFSE‐Panc02 cells were separately cultured with the pyroptotic tumor cells polarized BMDMs at the ratio of 1:1 for 4 h. After 4 h incubation, the percentage of BMDM cells that had engulfed CFSE‐KPC or CFSE‐Panc02 was measured by flow cytometry. The cells were labeled with F4/80 antibody to gate the BMDM group and the KPC and Panc02 cells were gated by CFSE. LPS‐induced M1‐like and IL‐4‐induced M2‐like macrophages treated with CFSE‐KPC or CFSE‐Panc02 was respectively set as the positive control and negative control for phagocytic clearance of cancer cells. To explore whether pyroptotic tumor cells could re‐educate the existing M2 to polarize to M1, IL‐4‐induced M2‐like macrophages were co‐cultured with pyroptotic tumor cells for 48 h. Then the expression of CD86 and MHC II on the macrophages was detected by flow cytometry. The CD11b antibody was used to gate the BMDM group. The IL‐4‐induced M2‐like macrophages were set as the negative group and the M2‐like macrophages after LPS (0.5 µg mL^−1^) treatment were set as the positive group. To analyze the CD8^+^ T cell proliferation and maturation, a total of 2 × 10^5^ T lymphocytes were incubated with pyroptotic cells for 48 h with the existence of BMDMs and BMDCs. Then the cells were collected and incubated with CD103 antibody to analyze the maturation. CFSE were used to gate the CD8^+^ T cell group and analyzed the proliferation of CD8^+^ T cells by flow cytometry. The weaker CFSE fluorescence represents the stronger T cell proliferation. To further evaluate the CD8^+^ T cell activation, a total of 2 × 10^5^ T lymphocytes were incubated with pyroptotic cells for 48 h with the existence of BMDMs and BMDCs or not. After 48 h incubation, CD8^+^ T cell activation was evaluated by analyzing granzyme B and perforin secretion by flow cytometry.

### Mice

The immunocompetent male C57BL/6 mice (4–6 weeks) were purchased from the Model Animal Research Center of Nanjing University and were maintained under a 12‐h light/12‐h dark cycle in pathogen‐free conditions.

### In Vivo Anti‐Tumor Therapy through PDT‐Aroused Pyroptosis

For the subcutaneous model, the KPC (or Panc02) cells were injected subcutaneously into the right flank of the male C57BL/6 mice at a density of 5 × 10^5^ (or 2 × 10^5^) suspended in 50 µL PBS per mouse. Ten days after incubation, mice were randomly divided into two groups, then PBS (25 µL) or TBD‐3C dissolved in PBS (1 mm, 25 µL) were intratumorally injected, respectively, and mice were all exposed to irradiation for 10 min at 40 mW cm^−2^ at 24 h after injection or under dark. Tumor volume was calculated by 1/2 × length × width^2^. The length means the longest diameter and the width means the perpendicular short diameter. At the end of the treatment course, the mice were sacrificed and the tumor samples were collected for further analysis. For the local and distant model, C57BL/6 male mice were subcutaneously inoculated with 5 × 10^5^ KPC‐Luc cells at the right flank to build a subcutaneous tumor model (local tumor) and injected with 5 × 10^5^ KPC‐Luc cells suspended in 25 µL mixed medium (Matrigel:PBS = 1:1) at the tail of the pancreas to build an orthotopic tumor model (distant tumor) with a sterile insulin needle. Seven days after incubation, mice were divided into two groups according to the subcutaneous and orthotopic tumor burdens which were measured by the IVIS. Specifically, D‐luciferin (200 mg kg^−1^) was intraperitoneal injected 20 min before imaging by Living Image software. Regions of interest were quantified as average radiance, represented by color bars. After the grouping, PBS (25 µL) or TBD‐3C dissolved in PBS (1 mm, 25 µL) was then intratumorally injected into “local tumor” and all mice were exposed to irradiation for 10 min at 40 mW cm^−2^ at 24 h after injection. Along with the administration, the subcutaneous and orthotopic tumor burdens were measured by the IVIS. At the end of the treatment course, the mice were sacrificed and the tumor samples were collected to evaluate the weights and perform other analyses.

### Immunohistochemistry (IHC)

Tumor tissues from mice were harvested at the end of the animal experiment and analyzed by IHC. First, the isolated tissues were stored in 10% neutral buffered formalin. After paraffin embedding, they were cut into 4 µm‐thick sections, placed on glass slides, and then deparaffinized after being baked at 68 °C for 90 min. Antigens were retrieved by boiling the sections in sodium citrate antigen retrieval solution for 10 min, then cooling at room temperature for half an hour. The samples were blocked in 3% BSA at room temperature for 30–60 min. After that, they were incubated with the target primary antibody at 4 °C overnight. The next day, the unbound primary antibody was washed with PBS and the secondary antibody conjugated with biotin was used to combine the primary antibody at room temperature for 60 min. The target protein was then visualized using a diaminobenzidine (DAB) chromogen kit, with brown staining representing a positive molecule. Finally, slides were stained with hematoxylin for nuclei for 3–5 min and the representative images were captured by optical microscope. The immunohistochemical results were further quantified by Image J software.

### ATP Activity Assay

ATP levels were measured using a firefly luciferase‐based ATP assay kit (Beyotime, China, S0026) according to the manufacturer's instructions. Briefly, 200 µL (20 mg tissue) lysate was added and a glass homogenizer was used to homogenize tissue. Centrifugation was performed at 4°C and 12 000 *g* for 5 min. The supernatant was taken for subsequent determination. The level of ATP was quantitatively determined by ATP bioluminescent assay kit according to manufacturer's instructions.

### Tumor Immune Microenvironment Assay by Flow Cytometry

At the end of in vivo therapy, the mice were sacrificed and the tumors were collected for flow cytometry analysis. The tumor tissues were separated into small fragments and were placed in RPMI 1640 containing CaCl_2_ (3 mm), DNase (10 µg mL^−1^), collagenase IV (1 mg mL^−1^), and 2% FBS at 200 rpm Shake for 60 min at 37 °C. The digested tissues were then milled into single cells using a 70‐µm cell strainer and washed with PBS 3 times. Then supernatants were removed and the cells were resuspended in a pre‐prepared 36% Percoll solution before being centrifuged at 600 *g* to remove non‐immune cells. Red blood cell lysis buffer was used to remove the red blood cells. To detect cytokines secreted by T cells, immune cells were stimulated by incubating with a leukocyte activation cocktail for 4 h at 37 °C. After that, cells were washed with PBS, blocked with anti‐mouse CD16/32 antibody for 30 min, and then stained with surface antibodies in the dark for 30 min at 4 °C. Cells were washed again with PBS, fixed with 4% paraformaldehyde, and then permeabilized with a fixation/perforation solution to simultaneously stain cells for intracellular molecules. Finally, the cells were resuspended in 400 µL PBS and analyzed by flow cytometry using a LSRFFortessa Cell Analyzer (BD Biosciences). These data were analyzed by FlowJo software.

### Mass Cytometry (CyTOF) Analysis of Immune Cells in Tumor Microenvironment

The immune cells in KPC‐bearing mice tumors were further analyzed by Mass cytometry (CyTOF). The methods of obtaining immune cells from tumor tissues are the same as mentioned in section “Tumor Immune Microenvironment Assay by Flow Cytometry.” The immune cells were incubated with an in‐house developed panel of mixed antibodies according to the standard protocol. The mixed antibody panel consists of 42 antibodies conjugated to different metals (Table S1, Supporting Information). After incubation, the CyTOF system (Helios, Fluidigm, San Francisco, CA, USA) was used to detect the metal signals to evaluate the expression of the conjugated target molecule. The types of immune cell were subjected to density clustering after they were identified using nonlinear dimensionality reduction [*t*‐distributed stochastic neighbor embedding (*t‐*SNE)].

### In Vivo Spleen Analysis

The spleens from mice were collected and prepared to single cell, followed by removal of red blood cells (RBCs) by Red blood cell lysis buffer. Then, the cells were stained with a LIVE/DEAD Fixable Violet Dead Cell Staining Kit, followed by blocking using anti‐CD16/32 mAb in PBS to block Fc receptors. For the analysis of memory T cells, cells were stained by CD3, CD4, CD8, CD44, and CD62L on ice for 30 min in the dark in PBS. Flow cytometry was performed by LSRFFortessa Cell Analyzer (BD Biosciences).

### Statistical Analysis

Quantitative data were presented as mean ± SD. The data were analyzed using GraphPad Prism software (GraphPad Inc., La Jolla, CA, USA; version 7.0). The differences were determined using Student's *t*‐test for two‐group comparisons. A *p*‐value less than 0.05 is considered statistically significant (*p*‐value: *p < 0.05, **p < 0.01, ***p < 0.001, and ****p < 0.0001).

## Conflict of Interest

The authors declare no conflict of interest.

## Author Contributions

M.W. and M.W. contributed equally to this work. M.W. and M.W. performed biochemistry, cell biology, and animal studies, and wrote the manuscript. X.L. performed the preparation and characterization of materials. S.S. and J.H. performed animal studies. B.L. and T.L conceived, designed, and supervised the project.

## Supporting information

Supporting Information

Supporting Information Movie S1

Supporting Information Movie S2

## Data Availability

Research data are not shared.
