# Supplementary material for: Pyroptosis Remodeling Tumor Microenvironment to Enhance Pancreatic Cancer Immunotherapy Driven by Membrane Anchoring Photosensitizer
Source: Adv Sci (Weinh). 2022 Aug 18;9(29):2202914. doi: 10.1002/advs.202202914 (PMC9561775; doi:10.1002/advs.202202914)
Supplement: Supplementary file 1 — Supporting Information [file ADVS-9-2202914-s003.pdf]

Supporting Information

Pyroptosis Remodeling Tumor Microenvironment to Enhance Pancreatic Cancer Immunotherapy Driven by Membrane Anchoring Photosensitizer

Meng Wang[+]*, Min Wu*[+]*, Xingang Liu, Shiyi Shao, Junmin Huang, Bin Liu*[*]*, Tingbo Liang*[*]

Supplementary Text

Instruments

The main instruments used in this project were as follows: CyTOF Helios system (Fluidigm, USA), LSRFFortessa Cell Analyzer (BD Biosciences, USA), Optical microscope (Leica, GER), LEICA SP8 confocal microscope (Leica, GER), ChemiScope (CliNX, CHN), SpectraMax M5 (Molecular Devices, USA), IVIS Spectrum Imaging System (Caliper Life Sciences, USA).

Cell culture

Murine KPC cell line, derived from spontaneous pancreatic cancer of genetically engineered (Kras$^{LSL\text{-}G12D}$, Trp53$^{LSL\text{-}R172H}$, and Pdx1-Cre) mice, was kindly gifted by Prof. Raghu Kalluri's laboratory from MD Anderson Cancer Center in USA. Panc02 cell line was obtained from American Type Culture Collection (ATCC). Luciferase- expressing KPC cell line (KPC-Luc) was isolated from KPC cell transfected with firefly luciferase report gene. The KPC and KPC-Luc cells were maintained in McCoy's 5A (Modified) Medium and the Panc02 cells were maintained in RPMI 1640 medium supplemented with 1% Penicillin/Streptomycin and 10% FBS. All cell lines were incubated in a humidified cell incubator at 37°C under an atmosphere of 5% CO_2.

Western Blot Analysis

To detect the FL-GSDMD protein after the treatment of TBD-3C (10 μM, 40 mW/cm^2) and detect the PRAP protein after TBD-3C (10 μM, 40 mW/cm^2) treatment or STS stimulation (2 μM for 4 h), the KPC and Panc02 cells were harvested after the corresponding treatment. Protein concentration was qualified by BCA assay. Then equal amount of protein was resolved in 10% SDS-PAGE, transferred to polyvinylidence fluoride (PVDF) membranes, blocked with 5% milk for 1 hour at room temperature and then incubated with primary antibodies at 4°C overnight. After washing with PBS three times, secondary antibody was incubated with the membrane. The results were detected and photographed by ChemiScope.

Isolation and differentiation of BMDM and BMDC

BMDM and BMDC were isolated from C57BL/6 mice. Briefly, the mice were sacrified and disinfected with 70% alcohol, the hip bones without skin and muscles were harvested and the

epiphyses of the bones were cut, and then the marrow were flushed into a 15 mL centrifuge tube using a 1 mL syringe. The resulting marrow were lysed with red blood cell lysis buffer and filtered through nylon mesh filters (70 μm), and centrifuged at 600×g and 4°C for 5 minutes. The obtained cells were diluted at a concentration of 1 × 10^6 cells/mL, and M-CSF was added at a final concentration of 20 ng/mL to differentiate BMDM or 10 ng/mL GM-CSF and 5 ng/mL IL-4 were added to differentiate BMDC. 10 mL of each type of cells were severally seeded onto cell culture-treated petri dish and incubated at 37°C with 5% CO_2 for 3 days. Replaced the medium and incubated for another 3 days. M1 and M2 macrophages were obtained after adding LPS (0.5 μg/mL) and IL-4 (20 ng/mL) separately for 48 h. The induced BMDMs were incubated with CD11b and F4/80 antibodies for 30 minutes. The expression of CD11b and F4/80 were measured using flow cytometer to confirm their phenotype. The BMDMs were incubated with CD86 and CD206 antibodies to show the differential expressions in the LPS-induced M1-like and IL-4-induced M2-like macrophages by flow cytometry to confirm their phenotype. M1-like and M2-like macrophages were defined as $CD86^{hi}$ $CD206^{lo}$ and $CD86^{lo}$ $CD206^{hi}$, respectively. The BMDCs were incubated with CD11c and MHC II antibodies to confirm their phenotype. The BMDMs and BMDCs were maintained in RPMI 1640 medium supplemented with 10% fetal bovine serum, 1% penicillin and streptomycin.

Isolation of T lymphocytes

T lymphocytes were obtained from the spleen of C57BL/6 mice. Spleen was mechanically dissociated into small pieces using a grind rod. The digested tissues were milled into single cells using a 70-μm cell strainer and washed with PBS for 3 times. Red blood cell lysis buffer was used to remove red blood cells. Then the $CD8^+$ T cell were purified using a mice $CD8^+$ T cell isolation kit (Miltenyibiotec, 130-096-495) according the manufacturer's instructions. The $CD8^+$ T cells were further stained using CD8-PE and CD3-FITC antibodies to confirm their phenotype. The isolated $CD8^+$ T cells were labeled with 4 μmol/L CFSE for 10 min at 37°C to obtain the CFSE-labeled $CD8^+$ T cells and were mixed with the Non-$CD8^+$ T cells in the column of the isolation kit for further use.

***In vivo* Biocompatibility assay**

At the end of the TBD-3C PDT treatment *in vivo*, the peripheral blood (1mL per mouse) was collected under anesthesia for blood biochemistry detection. The biochemistry indicators including creatine kinase (CK), creatinine (CREA), uric acid, urea were the liver and kidney

function index. The liver and kidney function tests were performed to investigate the biocompatibility of the TBD-3C PDT treatment.

And the major organs (hearts, livers, spleens, lungs and kidneys) of the mice were collected to evaluate the toxicity of the TBD-3C PDT treatment by H&E staining. For the H&E assay, the organs were fixed in 10% neutral buffered formalin for 24 h. After paraffin embedding, they were cut into 4 μm-thick sections and deparaffinized after being baked at 68°C for 90 minutes. The sections were dehydrated in graded ethanol and immersed in hematoxylin staining solution for 5 minutes. The color of the sections changed from blue to red 2 seconds after adding 50 uL 1% hydrochloric acid ethanol. The sections were then immersed in eosin staining solution for 5 minutes and washed with ddH_2O. Finally, the representative images were captured by ImageScope software.

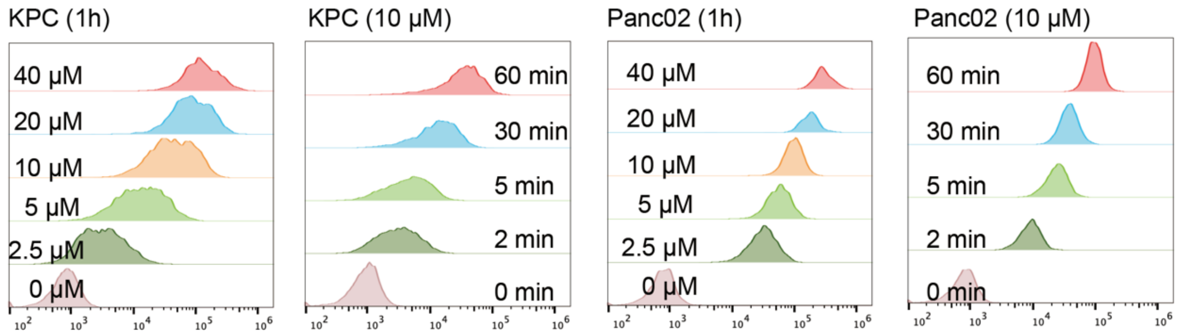

Figure S1. Exploration of labeling concentration and time. Flow cytometric analysis of KPC and Panc02 cells stained with different concentrations of TBD-3C for 1h and time-dependent TBD-3C (10 µM) labeling with KPC and Panc02 cells.

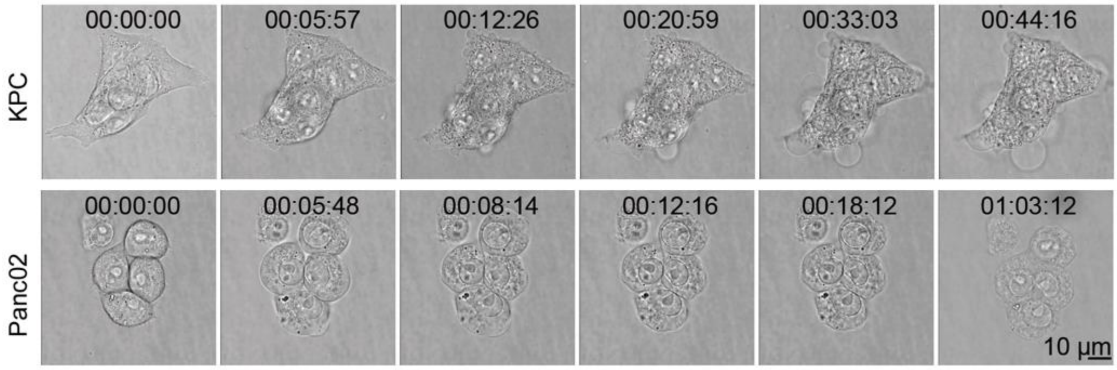

Figure S2. Time-lapse confocal microscopy images of co-culture of KPC and Panc02 cells after being treated with TBD-3C (10 μM) upon light illumination. Scale bars: 10 μm. (405 nm, 8%, Leica SP8 confocal microscope).

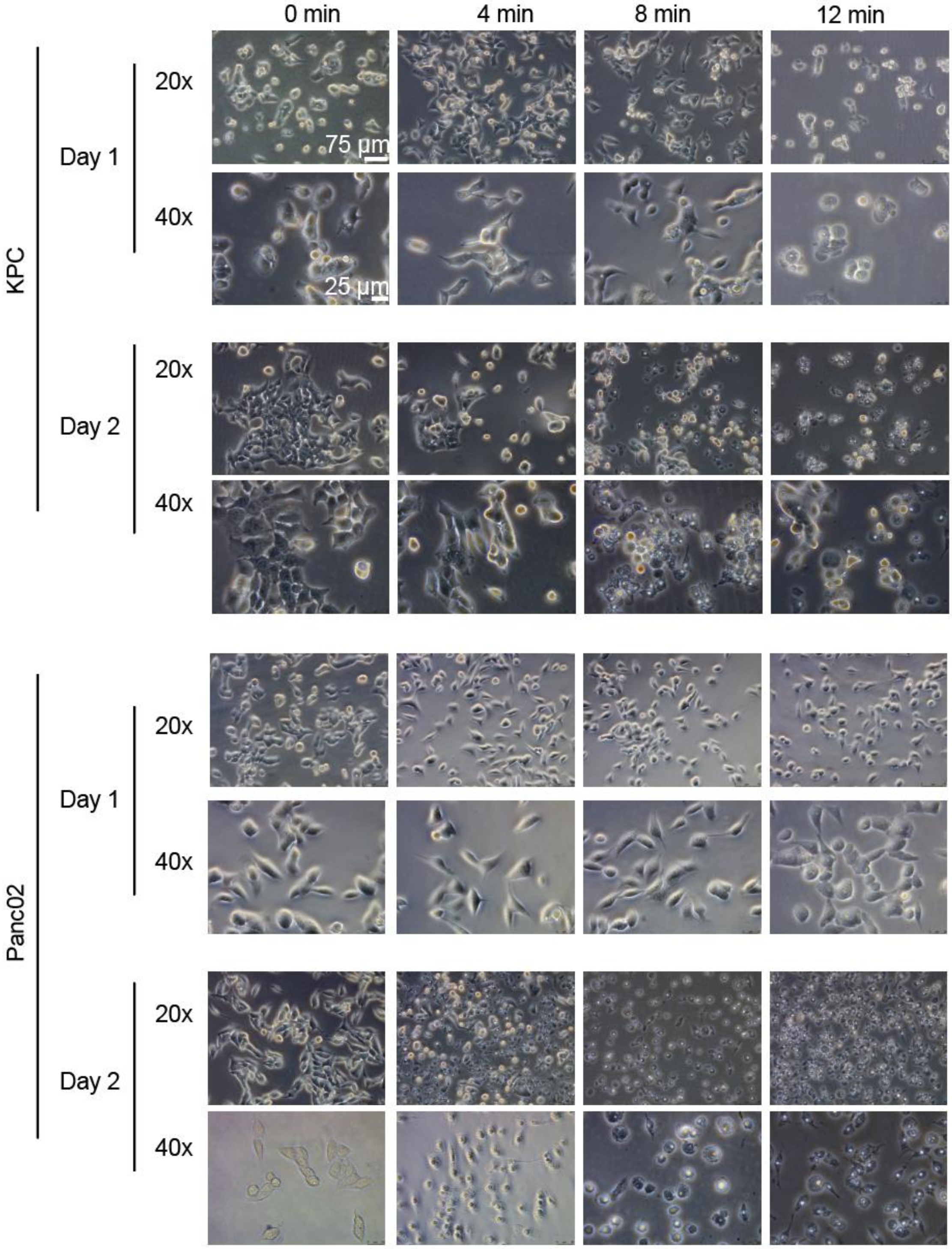

Figure S3. Microscopic images of KPC and Panc02 cells after being treated with TBD-3C (10 μM) upon light illumination at 40 mW/cm^2 for 0 min, 4 min, 8 min, and 12 min.

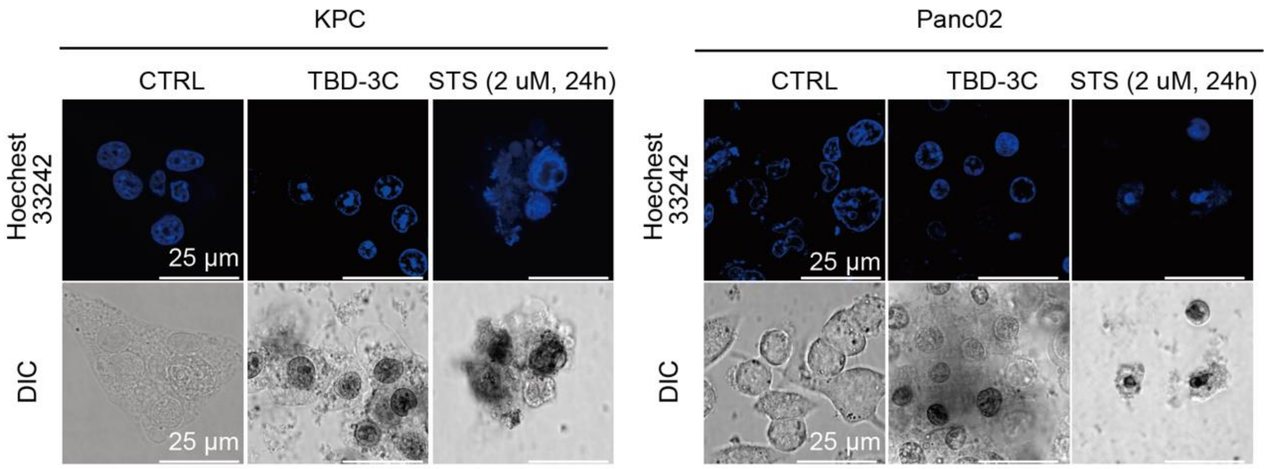

Figure S4. The effect on nuclear fragmentation of KPC and Panc02 cells after the treatment of TBD-3C (10 μM) upon irradiation at a power density of 40 mW/cm^2. The nucleus was stained with Hoechst 33342. STS (2 μM, 24 h) was used as a positive control to indicate the nuclear fragmentation. Scale bars: 25 μm. DIC: differential interference contrast image.

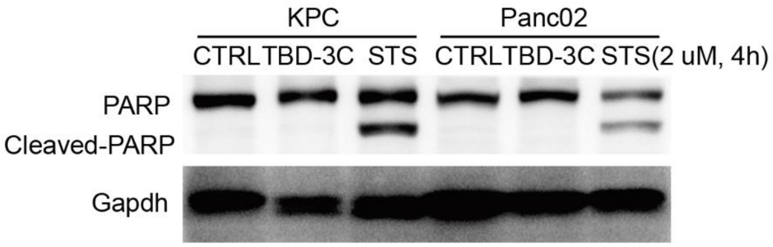

Figure S5. The effect on PARP cleavage of KPC and Panc02 cells after the treatment of TBD-3C (10 μM) upon irradiation at a power density of 40 mW/cm^2 compared to STS stimulation (2 μM for 4 h).

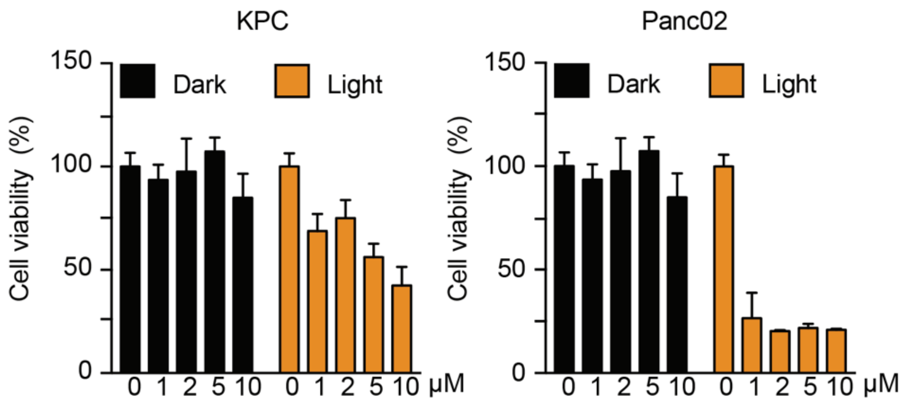

Figure S6. Cell viability of KPC and Panc02 cells after being treated with TBD-3C upon light illumination at 40 mW/cm^2 for 10 min or under dark.

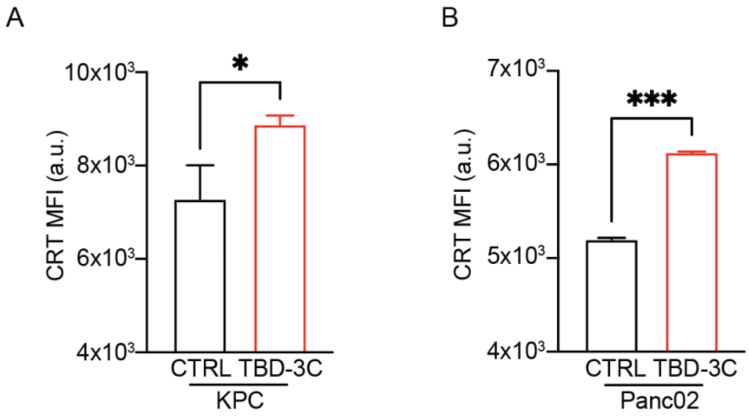

Figure S7. Flow cytometry assessment of CRT exposure on KPC cells (A) and Panc02 cells (B) treated with TBD-3C upon light illumination with Alexa Fluor 647-conjugated anti-CRT antibody (ab196159, Abcam). The cells treated with PBS were set as the control group. Data are presented as the mean + SD. Statistical analysis was performed using the Student's *t*-test (*$p < 0.05$, ***$p < 0.001$) ($n = 3$). (MFI, Mean Fluorescence Intensity).

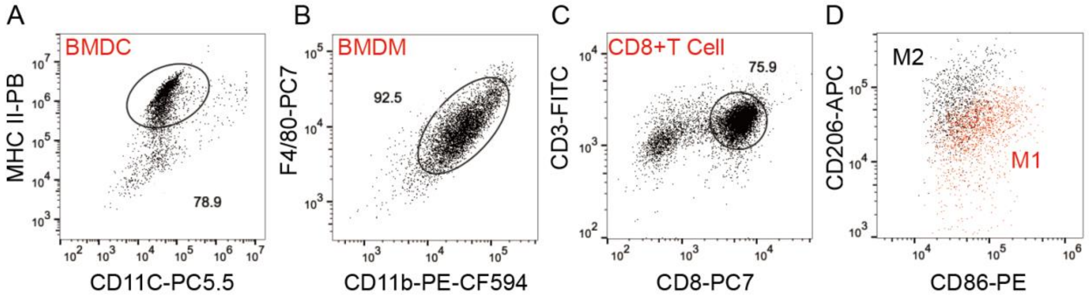

Figure S8. (A-D) Identification of BMDC **(A)**, M0 **(B)**, CD8$^+$ T cell subsets **(C)**, M1-like, and M2-like macrophages **(D)**.

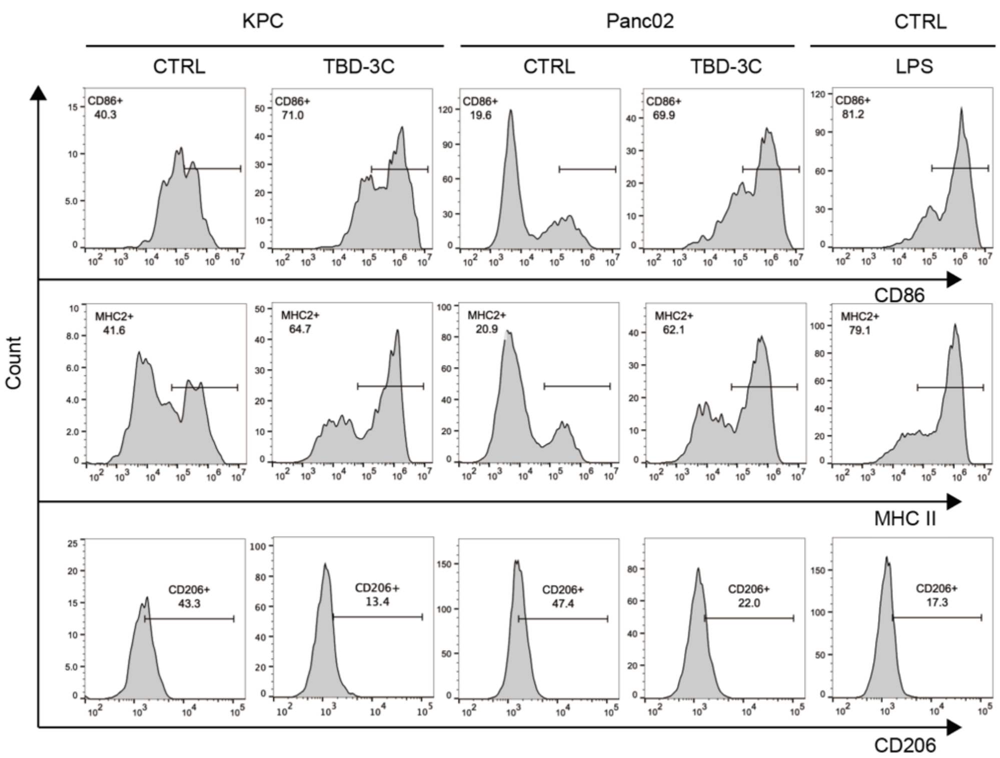

Figure S9. The representative cytometry patterns related to **Figure 2A, B, C.**

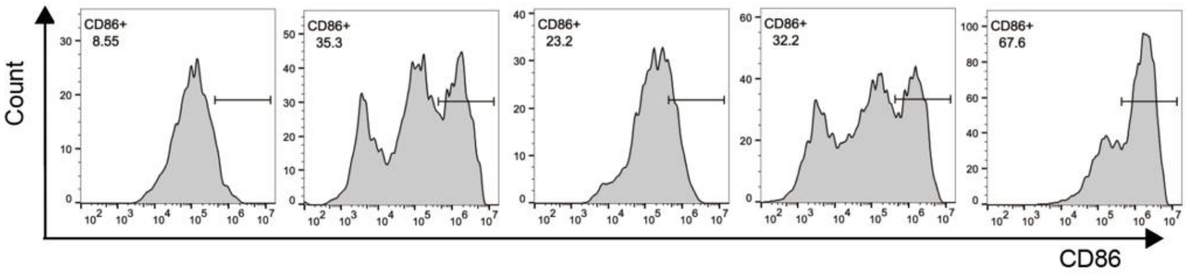

Figure S10. The representative cytometry patterns related to **Figure 2D**.

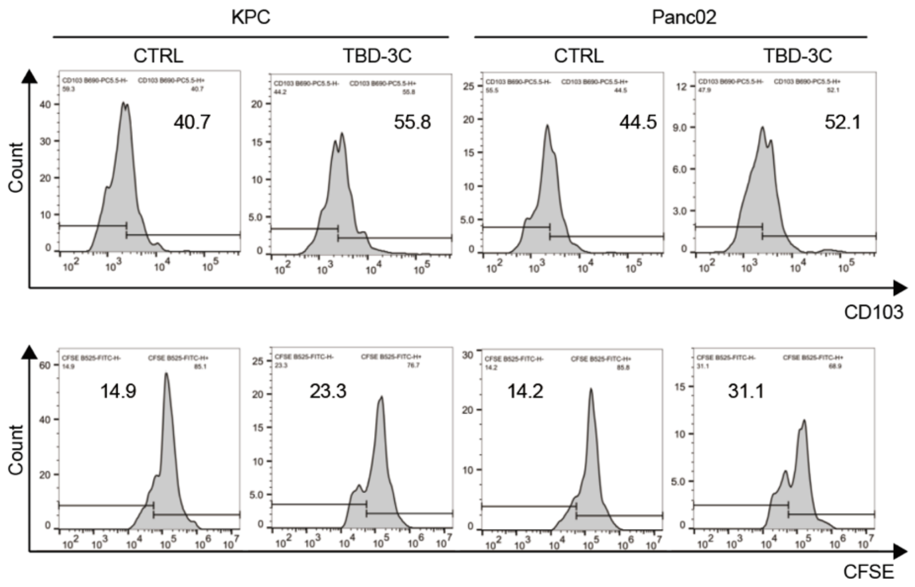

Figure S11. The representative cytometry patterns related to **Figure 2I and 2J**.

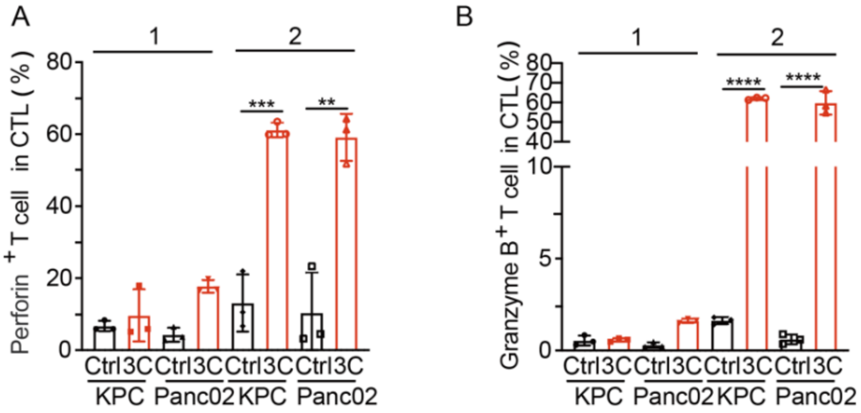

Figure S12. Assessment of perforin (A) and granzyme B (B) markers on CTLs after the pyroptotic cells (KPC and Panc02) co-culture with (2) or without (1) BMDM and BMDC cells. Statistical analysis was performed by Student's *t*-test (*$p < 0.05$, **$p < 0.01$, and ***$p < 0.001$) (n = 3).

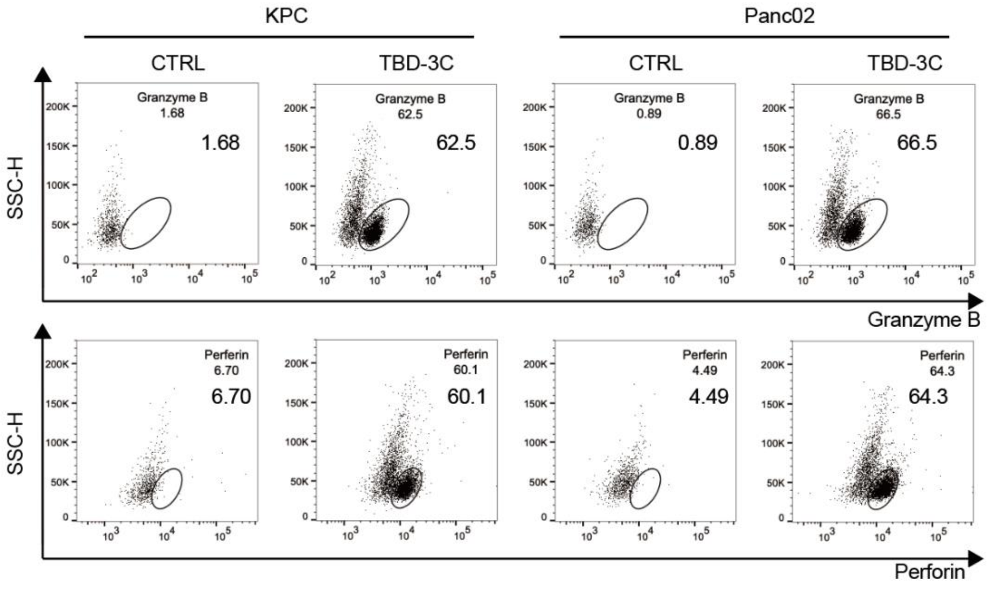

Figure S13. The representative cytometry patterns related to **Figure 2K and 2L.**

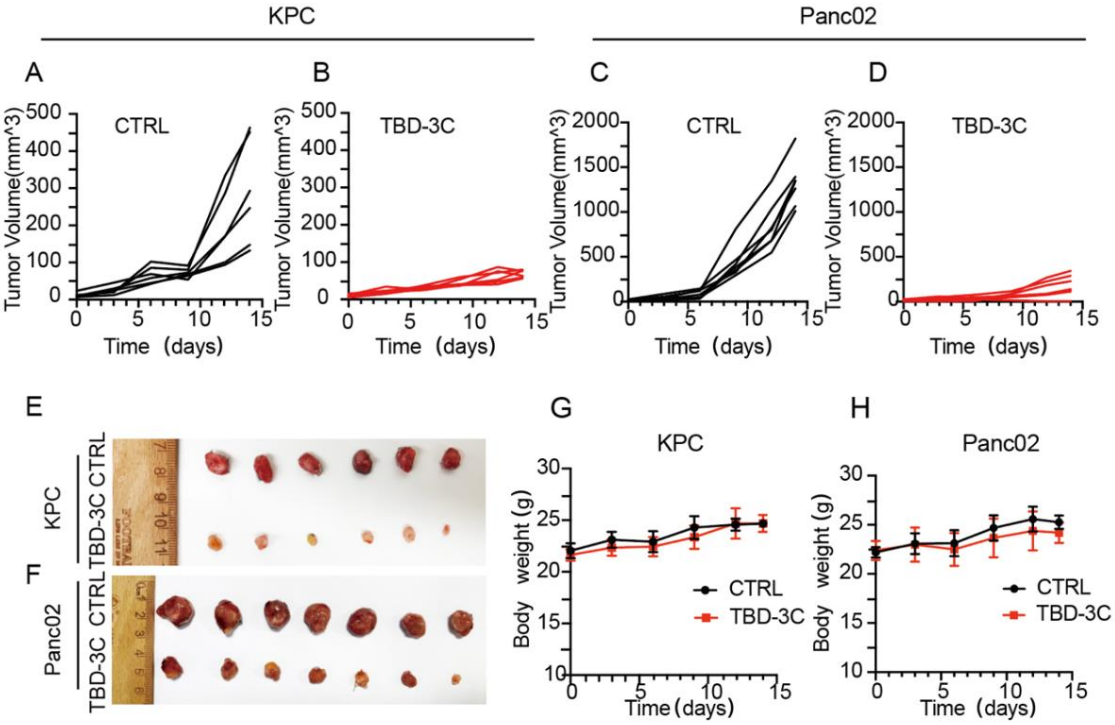

Figure S14. ***In vivo*** **antitumor effects of localized delivery of TBD-3C upon light irradiation in KPC and Panc02 tumor-bearing mice. (A-D)** Tumor growth curves in the different groups in KPC model **(A and B)** and Panc02 model **(C and D)**. **(E and F)** Images of the tumor in KPC model **(E)** and Panc02 model **(F)**. **(G and H)** Body weights in the different groups in KPC model **(G)** and Panc02 model **(H)**.

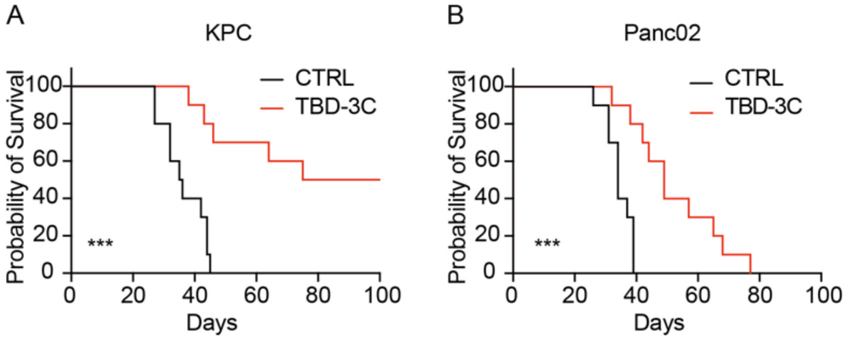

Figure S15. Survival curves of mice after various treatments in KPC model **(A)** and Panc02 model **(B)**. Kaplan-Meier survival curves were constructed using the log-rank test for significance between the control group and TBD-3C PDT group. (***$p < 0.001$) (n = 10).

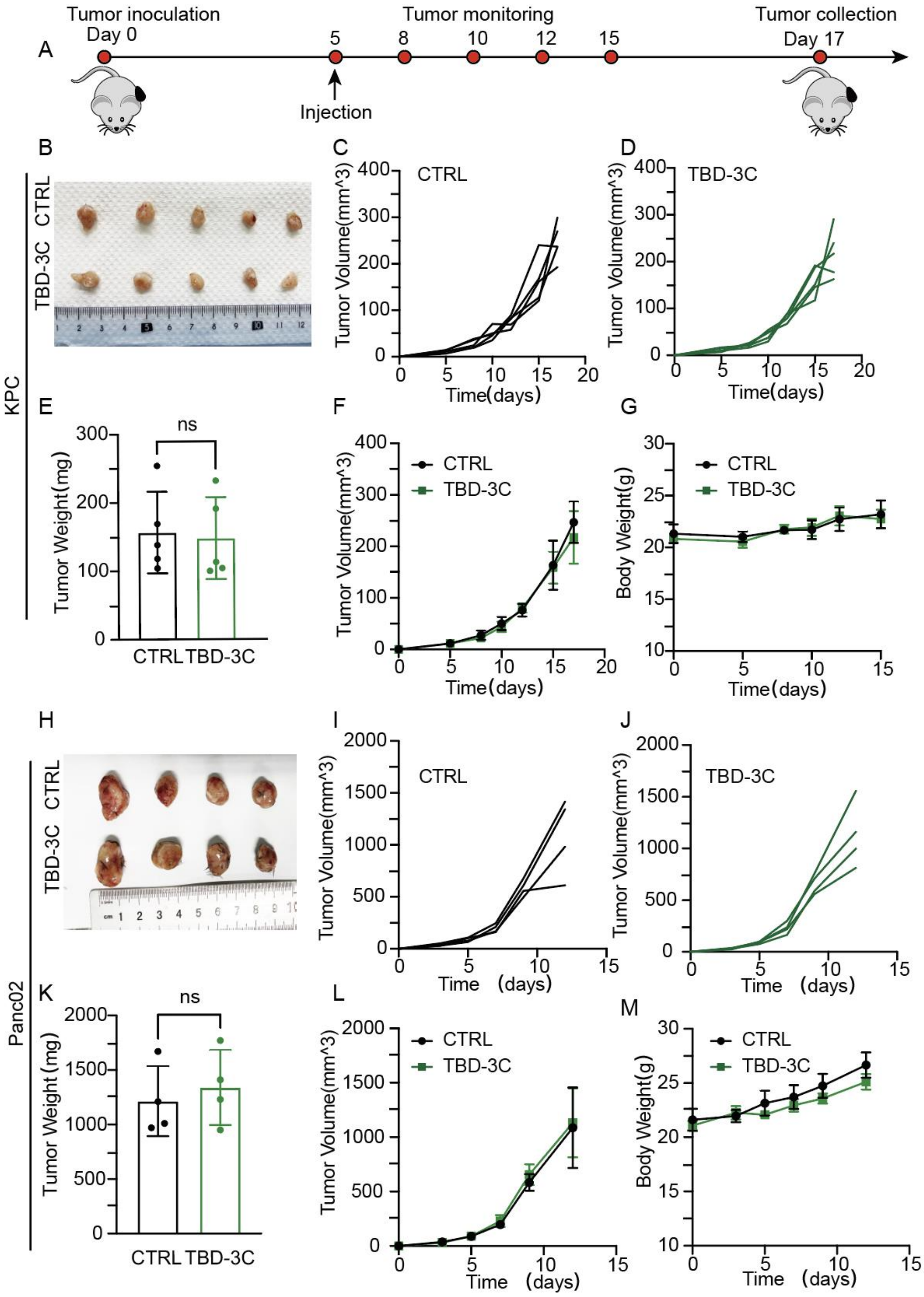

Figure S16. *In vivo* antitumor effects of localized delivery of TBD-3C without irradiation in KPC and Panc02 tumor-bearing mice. (A) Protocol for tumor occurrence assay. C57 mice were subcutaneously inoculated with 5 × 10^5 KPC or Panc02 cells on day 0. PBS or TBC-3C was then intratumorally injected on day 5 without irradiation subsequently. Images of the tumor in the different groups at the end of antitumor evaluation in KPC model **(B)** and Panc02 model **(H)**. The weights of tumor **(E, K)** and tumor growth curves **(C-L)** in the different groups. Body weights in the different groups in the KPC model **(G)** and Panc02 model **(M)**.

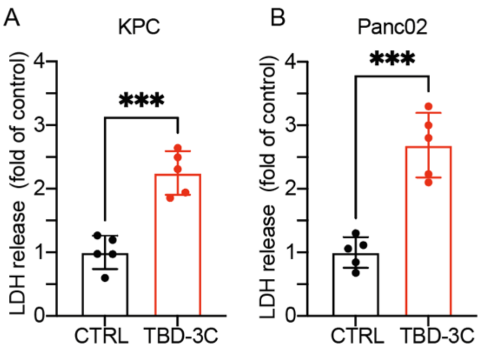

Figure S17. LDH release examination in tumors in KPC model **(A)** and Panc02 model **(B)** after different treatments. Data are presented as the mean ± SD. Statistical analysis was performed by Student's *t*-test (***$p < 0.001$) (n = 5).

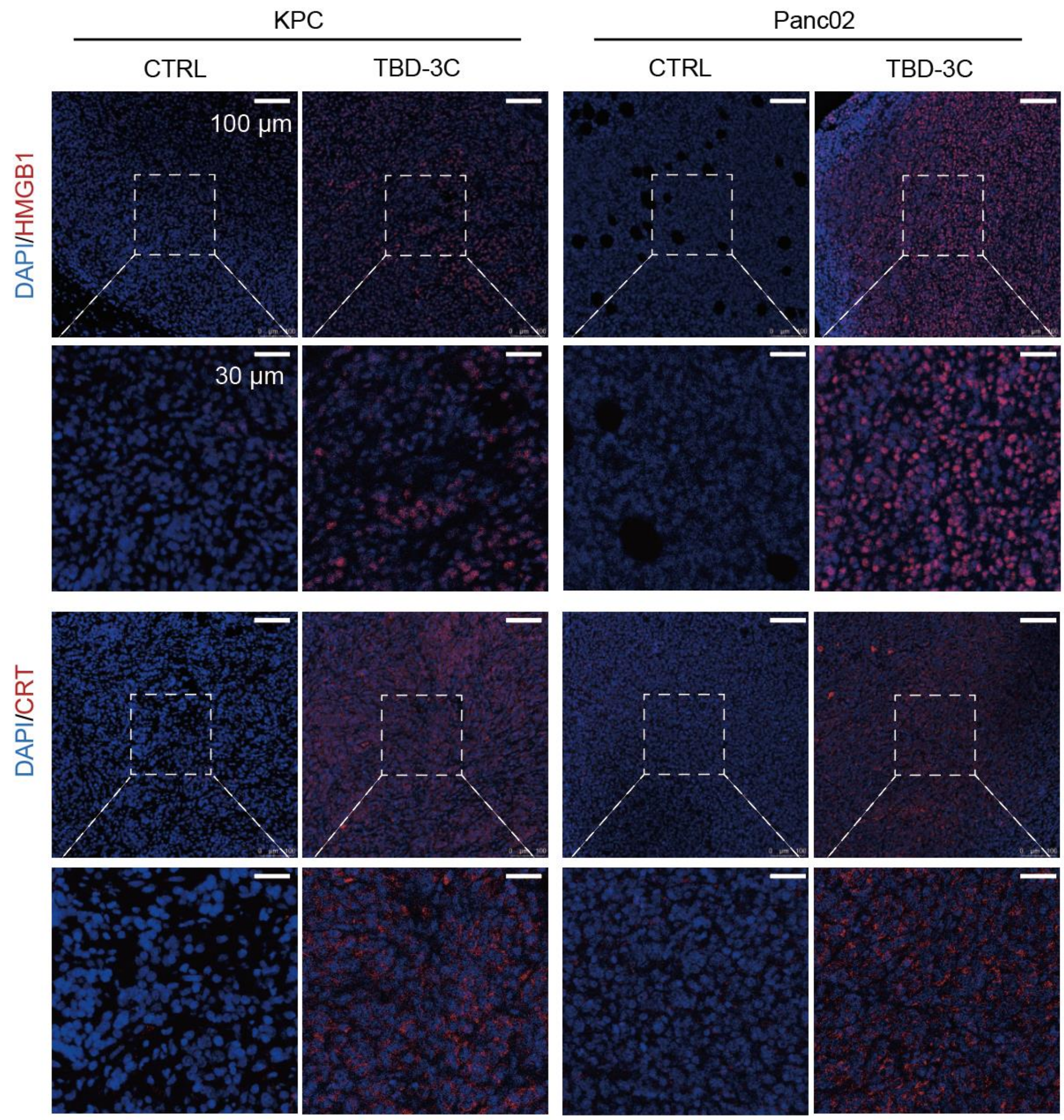

Figure S18. Immunofluorescence analysis of HMGB1 and CRT expression in KPC model and Panc02 model after treatments. Red represent the target protein (HMGB1 and CRT), and blue represent the nuclei, respectively.

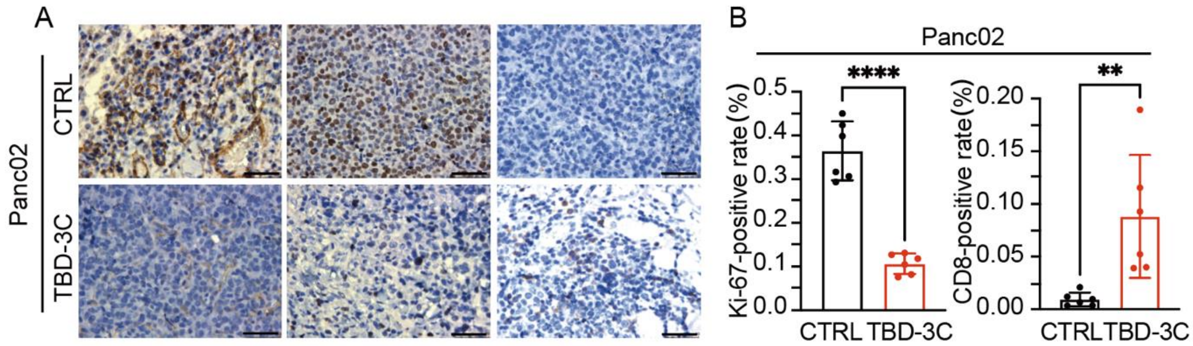

Figure S19. (A) Pathology studies show α-SMA, Ki-67, and CD8 expression in the mice in different groups in Panc02 model. The bars represent 50 μm. **(B)** Ki-67 and CD8 positivity analyses in Panc02 model (n = 5). Data are presented as the mean ± SD. Statistical analysis was performed by Student's *t*-test (***p* < 0.01, and *****p* < 0.0001).

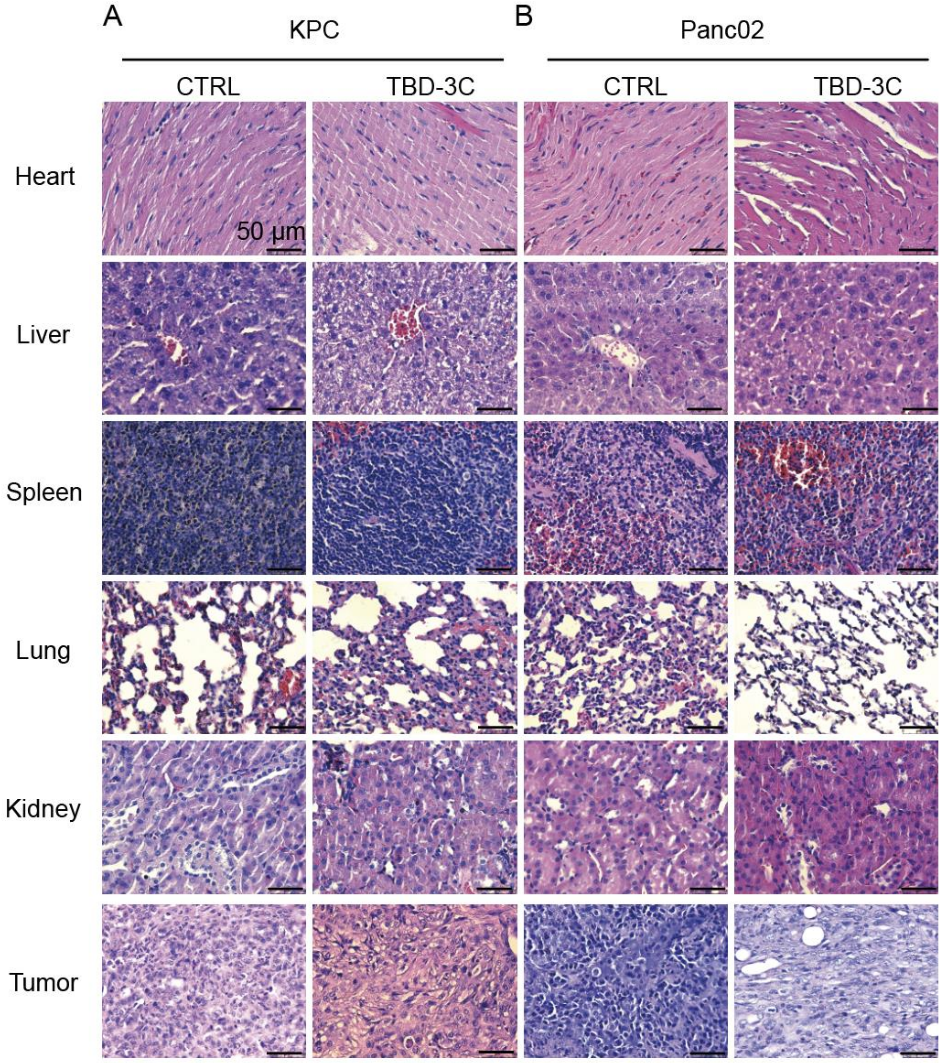

Figure S20. Pathology studies of the organs in the mice treated with PBS and TBD-3C (under light) in the KPC model **(A)** and Panc02 model **(B)**.

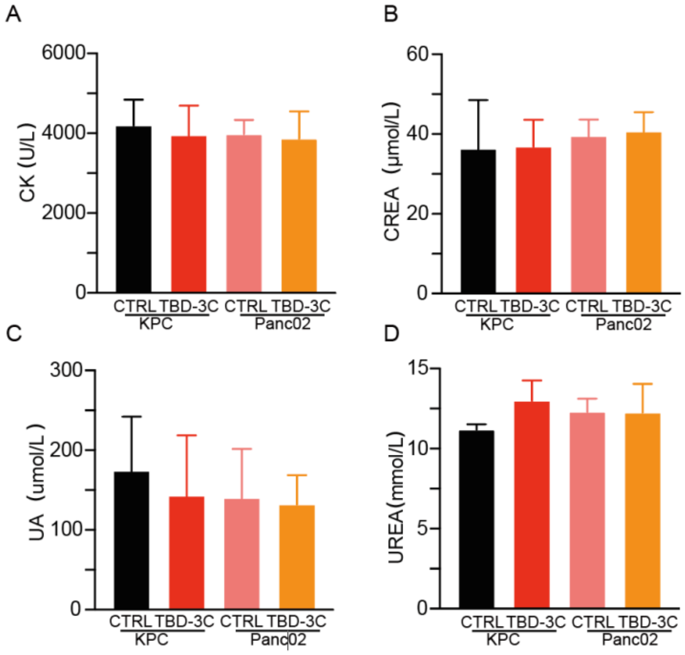

Figure S21. Results of the blood metabolic in mice treated with PBS and TBD-3C (under light).

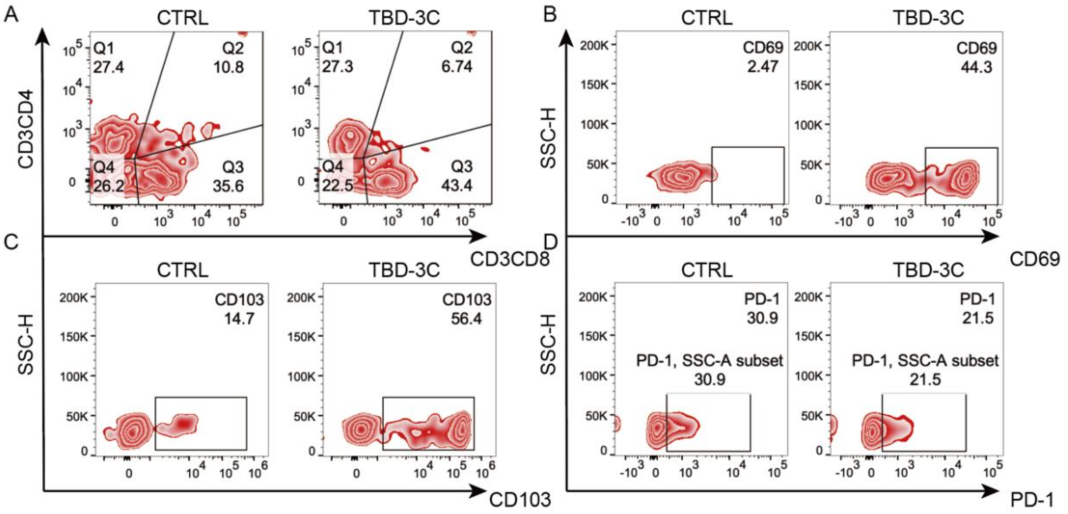

Figure S22. The representative cytometry patterns related to **Figure 3H-3K**, respectively.

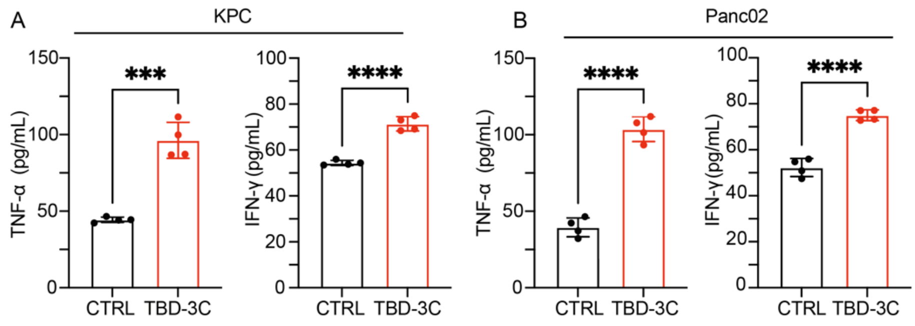

Figure S23. TNF-α and IFN-γ levels in KPC **(A)** and Panc02 **(B)** mice serum after different treatments. Data are presented as the mean ± SD. Statistical analysis was performed using the Student's *t*-test (***p < 0.001, and ****p < 0.0001) (n = 4).

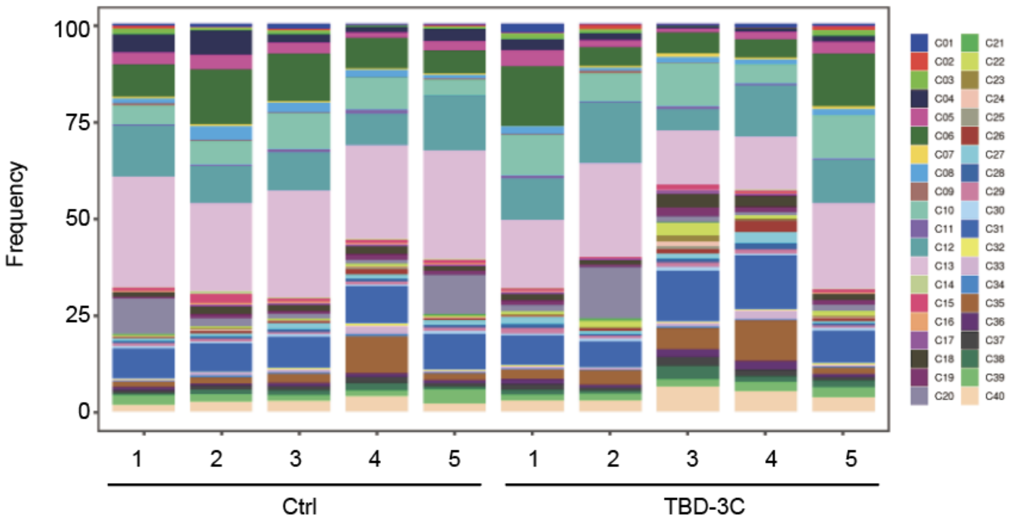

Figure S24. Representative images of cell proportion in different groups.

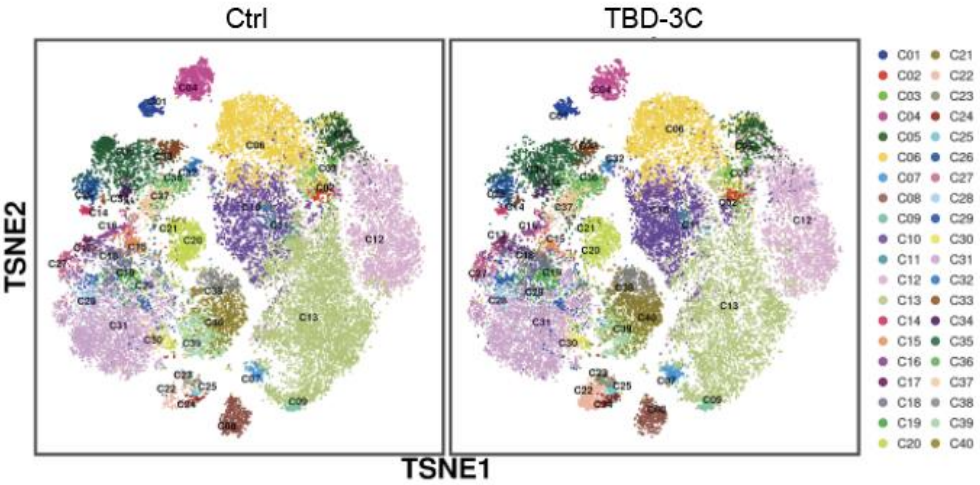

Figure S25. The t-SNE maps colored by 40 clusters from different groups.

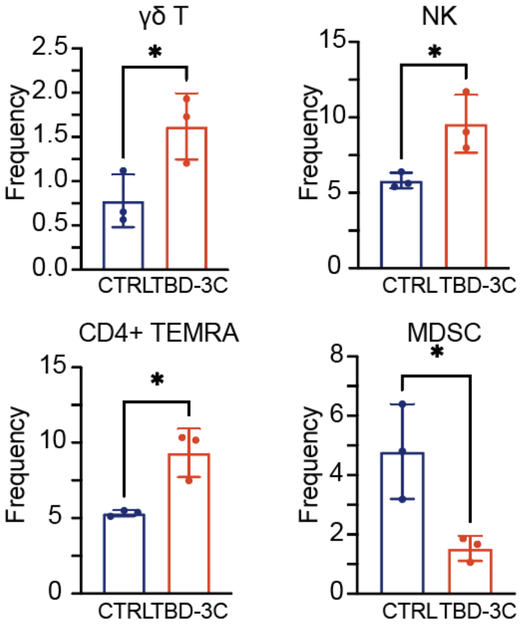

Figure S26. The frequency of γδT cell, NK cell, $CD4^+$ TEMRA, and MDSC in different groups. Data are presented as the mean ± SD. Statistical analysis was performed by Student's *t*-test (*$p < 0.05$) (n = 3).

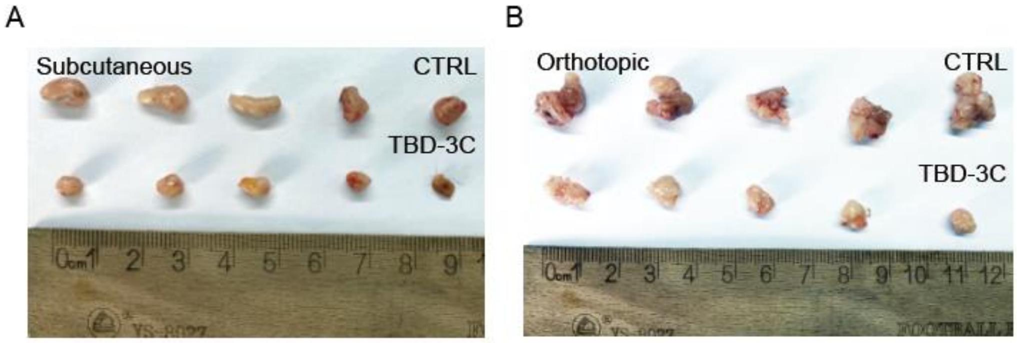

Figure S27. Images of the tumor in the different groups in KPC subcutaneous model **(A)** and KPC orthotopic model **(B).**

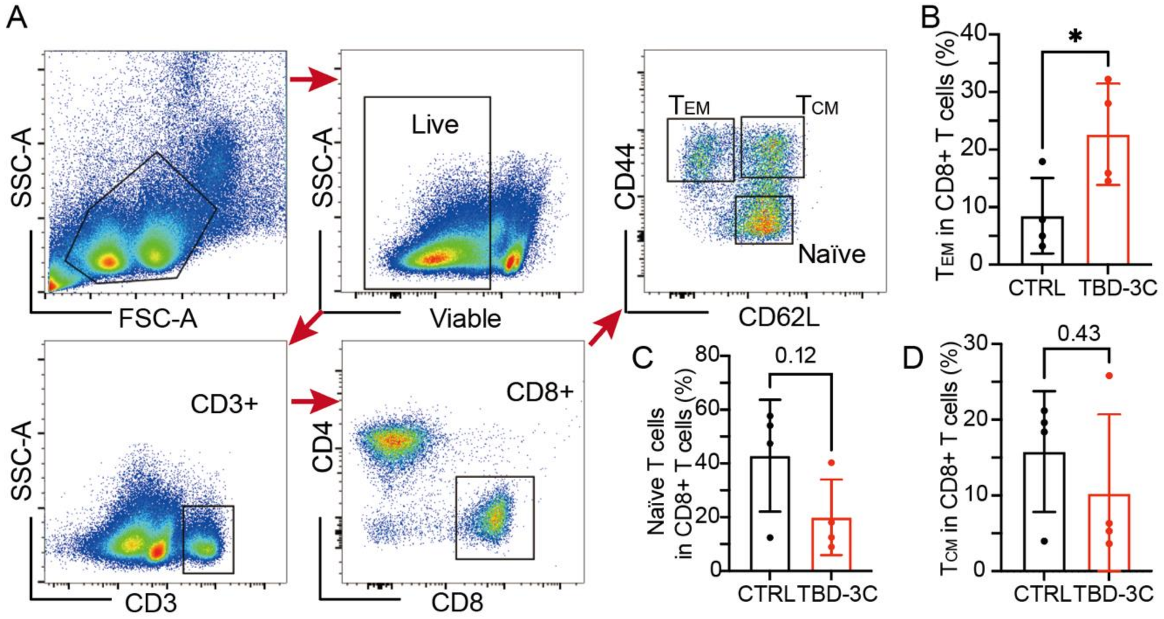

Figure S28. **(A)** Gating strategy of FACS analysis for detecting naïve T cells, T_{EM}, and T_{CM} in the CD8+ T cells from the spleen of the tumor-bearing mice with different treatments. **(B-D)** Statistic results for the proportions of T_{EM} **(B)**, naïve T cells **(C)** and T_{CM} **(D)** in the CD8+ T cells of each group. Data are presented as the mean ± SD. Statistical analysis was performed using the Student's *t*-test (*p < 0.05) (n = 4).

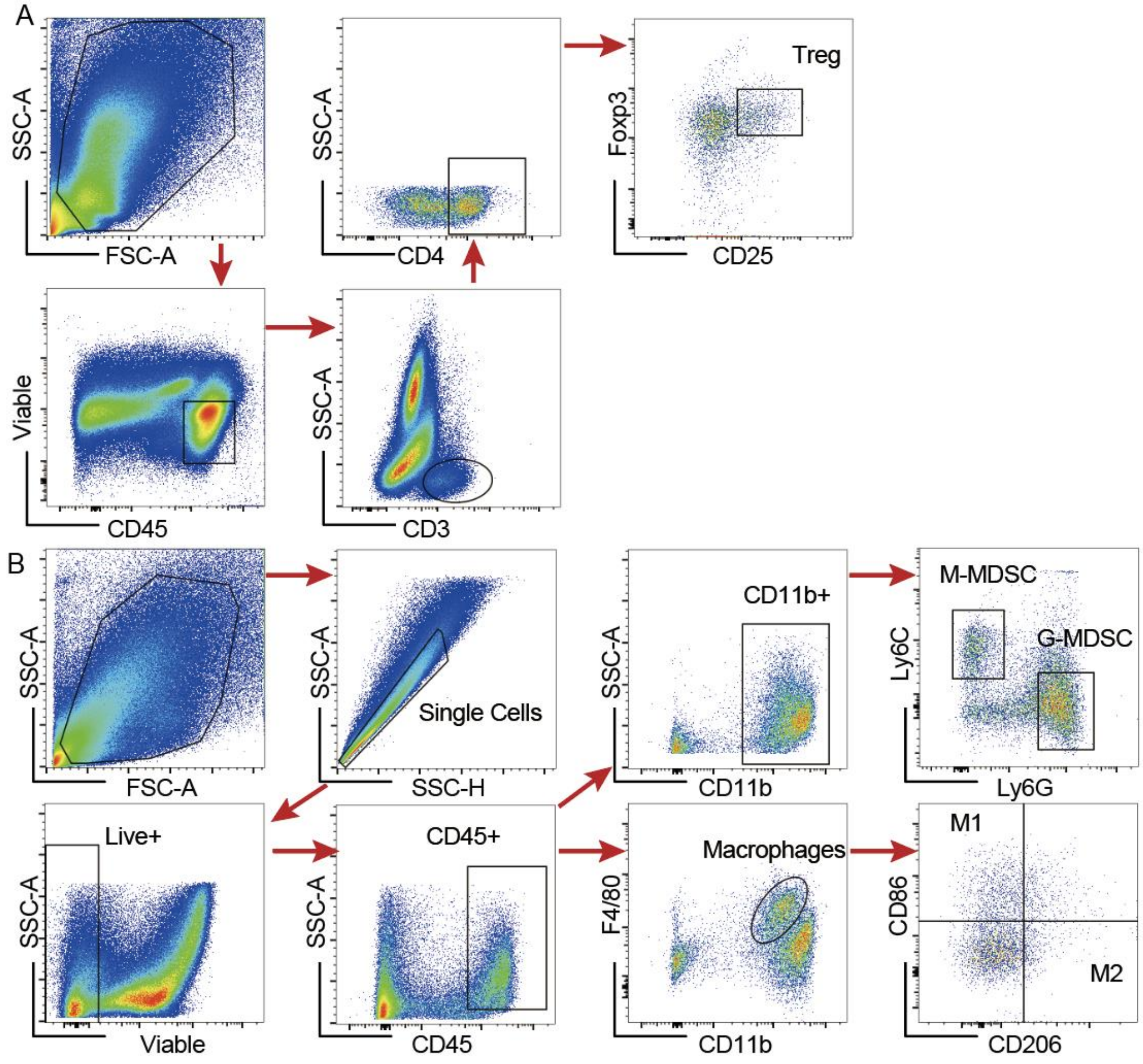

Figure S29. (A) Gating strategy of FACS analysis for detecting Tregs in the TME of distant tumor from the tumor-bearing mice with different treatments in KPC model. **(B)** Gating strategy of FACS analysis for detecting M-MDSC, G-MDSC, M1 macrophages, and M2 macrophages in the TME of distant tumor from the tumor-bearing mice with different treatments in KPC model.

Table S1: List of Antibodies in CyTOF

List	Label	Marker	Clone	Company	Code
1	89Y	CD45	30-F11	Biolegend	103102
2	115In	CD3e	145-2C11	Biolegend	100302
3	139La	Ki67	SolA15	eBioscience	14-5698-82
4	141Pr	CD24	M1/69	Biolegend	101802
5	142Nd	MHC II	M5/114.15.2	Biolegend	107602
6	143Nd	CD45R_B220	RA3-6B2	Biolegend	103202
7	144Nd	CD183_CXCR3	CXCR3-173	Biolegend	126502
8	145Nd	CD163	S15049I	Biolegend	155302
9	146Nd	CD279_PD1	29F.1A12	Biolegend	135202
10	147Sm	CD80	16-10A1	Biolegend	104702
11	148Nd	Ly6C	HK1.4	Biolegend	128002
12	149Sm	CD19	6D5	Biolegend	115502
13	150Nd	IL10	JES5-16E3	Biolegend	505002
14	151Eu	CD62L	MEL-14	Biolegend	104402
15	152Sm	CD11c	N418	Biolegend	117302
16	153Eu	CD44	IM7	Biolegend	103002
17	154Sm	Tbet	4B10	BioLegend	644802
18	155Gd	CD223_Lag3	C9B7W	Biolegend	125202
19	156Gd	CD14	Sa14-2	Biolegend	123302
20	157Gd	CD366_Tim3	RMT 3-23	Biolegend	119702
21	158Gd	TCR γ/δ	GL3	Biolegend	118140
22	159Tb	F4/80	C1:A3-1	BioRAD	MCA497G
23	160Gd	CD86	GL-1	Biolegend	105002
24	161Dy	CD274_PDL1	10F.9G2	Biolegend	124302
25	162Dy	CD69	H1.2F3	Biolegend	104502
26	163Dy	CD25	3C7	Biolegend	101902
27	164Dy	RORγ	600214	R&D	MAB6109
28	165Ho	IFNγ	XMG1.2	Bio-Xcell	BE0055
29	166Er	Ly6G	1A8	Biolegend	127602
30	167Er	CD206	C068C2	Biolegend	141702

31	168Er	FoxP3	FJK-16s	eBioscience	14-5773-82
32	169Tm	CD127_IL7Ra	A7R34	Biolegend	135002
33	170Er	CD161_NK1.1	PK136	Biolegend	108702
34	171Yb	GATA3	TWAJ	eBioscience	14-9966-82
35	172Yb	Perforin	OMAK-D	Fluidigm	3172018B
36	173Yb	Granzyme B	GB11	Fluidigm	3173006B
37	174Yb	CD152_CTLA4	UC10-4B9	Biolegend	106302
38	175Lu	IL2	JES6-5H4	Biolegend	503802
39	176Yb	TNFα	MP6-XT22	Biolegend	506302
40	197Au	CD4	RM4-5	Biolegend	100520
41	198Pt	CD8a	53-6.7	Biolegend	100746
42	209Bi	CD11b	M1/70	BioLegend	101202

Table S2: Definition of Cluster in CyTOF.

Cluster	Definition-1	Definition-2	Cluster	Definition-1	Definition-2
1	Macrophage		21	other	
2	Macrophage	M2	22	γδ T	
3	Macrophage	M1	23	γδ T	
4	Granulocytes	MDSC	24	γδ T	
5	Macrophage	M1	25	γδ T	
6	Macrophage		26	CD8+T	
7	DC		27	CD4+T	CD4+TEMRA
8	B cell		28	CD4+T	CD4+TEMRA
9	DC		29	CD4+T	CD4+TEMRA
10	Macrophage	M1	30	other	
11	Macrophage	M1	31	CD4+T	Treg
12	DC		32	Monocytes	
13	DC		33	CD8+T	
14	CD8+T	CD44-CD62-	34	CD8+T	CD44-CD62-
15	CD4+T	CD44-CD62-	35	CD8+T	
16	CD4+T	CD44-CD62-	36	Monocytes	
17	CD4+T	CD4+TEMRA	37	Monocytes	
18	CD4+T	CD4+TEMRA	38	NK	
19	CD4+T	CD4+TEMRA	39	NK	
20	other		40	NK	
